# LRRK2 kinase activity restricts NRF2-dependent mitochondrial protection in microglia

**DOI:** 10.1093/jimmun/vkaf215

**Published:** 2025-09-04

**Authors:** Chi G Weindel, Aja K Coleman, Lily M Ellzey, Sandeep Kumar, Sara L Chaisson, Jacob R Davis, Kristin L Patrick, Robert O Watson

**Affiliations:** Department of Microbial Pathogenesis and Immunology, Texas A&M Health Science Center, Texas A&M School of Medicine, Bryan, TX, United States; Department of Microbiology and Immunology, Tulane University School of Medicine, New Orleans, LA, United States; Department of Microbial Pathogenesis and Immunology, Texas A&M Health Science Center, Texas A&M School of Medicine, Bryan, TX, United States; Department of Pathology, Microbiology, and Immunology, Vanderbilt University Medical Center, Nashville, TN, United States; Department of Microbiology and Immunology, Tulane University School of Medicine, New Orleans, LA, United States; Department of Microbiology and Immunology, Tulane University School of Medicine, New Orleans, LA, United States; Department of Microbial Pathogenesis and Immunology, Texas A&M Health Science Center, Texas A&M School of Medicine, Bryan, TX, United States; Department of Microbial Pathogenesis and Immunology, Texas A&M Health Science Center, Texas A&M School of Medicine, Bryan, TX, United States; Department of Pathology, Microbiology, and Immunology, Vanderbilt University Medical Center, Nashville, TN, United States; Department of Microbial Pathogenesis and Immunology, Texas A&M Health Science Center, Texas A&M School of Medicine, Bryan, TX, United States; Department of Medicine, Division of Infectious Diseases, Vanderbilt University Medical Center, Nashville, TN, United States

**Keywords:** LRRK2 kinase inhibitors, NRF2, mitochondria, type I IFN, tissue-specific macrophages

## Abstract

Mounting evidence supports a critical role for central nervous system (CNS) glial cells in neuroinflammation and neurodegenerative diseases, including Alzheimer’s disease (AD), Parkinson’s Disease (PD), Multiple Sclerosis (MS), as well as neurovascular ischemic stroke. Previously, we found that loss of the PD-associated gene leucine-rich repeat kinase 2 (*Lrrk2*) in macrophages, peripheral innate immune cells, induced mitochondrial stress and elevated basal expression of type I interferon (IFN) stimulated genes (ISGs) due to chronic mitochondrial DNA engagement with the cGAS/STING DNA sensing pathway. Here we report that loss of LRRK2 results in a paradoxical response in microglial cells, a CNS-specific macrophage population. In primary murine microglia and microglial cell lines, loss of *Lrrk2* reduces tonic IFN signaling leading to a reduction in ISG expression. Consistent with reduced type I IFN, mitochondria from *Lrrk2* KO microglia are protected from stress and have elevated metabolism. These protective phenotypes involve upregulation of NRF2, an important transcription factor in the response to oxidative stress and are restricted by LRRK2 kinase activity. Collectively, these findings illustrate a dichotomous role for LRRK2 within different immune cell populations and give insight into the fundamental differences between immune regulation in the CNS and the periphery.

## Introduction

Microglia are slow dividing long-lived resident macrophage populations of the central nervous system (CNS)^1^^,^^2^ that develop from yolk sac myeloid hematopoietic precursors and travel to the brain prior to closure of the blood brain barrier.^3–6^ As an innate immune population, microglia act as the first line of defense against invading pathogens and cellular damage through multiple processes including phagocytosis, neuron pruning, cytokine release, and direct cell-cell communication.^7–12^ The tight regulation of these processes is necessary to prevent neuroinflammation, a hallmark of neurodegenerative diseases of the CNS.^13–16^

The type I interferon (IFN) response has gained recent interest for its role in both maintaining neuron health as well as promoting inflammation. Signaling through the IFNα/β receptor (IFNAR) has been shown to be critical for the development of healthy neurons, as cell-type specific loss of IFNAR signaling results in Lewy body accumulation, α-synuclein aggregation, and a PD-like phenotype due to a blockade of neuronal autophagy.^17^ Microglial type I IFN responses also play crucial roles in neurodevelopment, where IFNAR signaling facilitates phagocytosis and clearance of damaged neurons.^18^ Although type I IFN is critical for the healthy development of the CNS, IFNAR signaling can act as a double-edged sword to promote neurodegeneration. For example, in the 5xFAD model of amyloid β-induced Alzheimer’s, blocking IFNAR in CNS cell populations was protective against memory loss and synaptic damage.^19^^,^^20^ In naturally aging brains, type I IFN signatures in microglial cells are enhanced^21^ and are associated with low level inflammation, bystander cell activation, and microgliosis.^22^ Given what we know, increased IFN signatures could be a product of elevated microglial phagocytic activity and protection, or aberrant inflammation, bystander cell activation, and CNS damage. Thus, there is a critical need to better understand the regulatory nodes that govern protective vs. pathogenic type I IFN responses in the brain, and how this regulation drives the maintenance of healthy glia and neurons while restricting neuroinflammation.

In the peripheral immune system, the tempering of type I IFN responses is a critical means to restrict inflammation and prevent interferonopathies and autoimmunity. One well-known restrictive pathway is the NRF2-mediated redox response. Classically, NRF2 is a transcription factor that upregulates antioxidant proteins to protect against oxidative stress. NRF2 has also been shown to regulate immune signaling; it is a negative regulator of type IFN during viral infection^23^^,^^24^ and can restrict IFN-β activation and inflammation following LPS stimulation or sepsis.^25–27^ NRF2 restricts the type I IFN response at several nodes, including inhibiting dimerization of the transcription factor IRF3^28–30^ and reducing STING expression and mRNA stability in human cells.^31^ Proteins upregulated by NRF2 during oxidative stress such as HMOX1 also have regulatory effects on the type I IFN response through degradation of transcription factors IRF3/IRF7 via autophagy,^32^ suggesting a complex interplay between the two pathways. Less is known about the connection between NRF2 and type I IFN responses in the brain. It has been shown that mice lacking NRF2 have neuroinflammation with astrogliosis,^33^ and NRF2 modulation impacts neuroinflammation in several PD models.^34^^,^^35^ Despite the intriguing connections between NRF2 and brain health, the role of NRF2 in glial cell inflammation remains under studied.

LRRK2 is a multifunctional PD-associated kinase expressed in neurons and immune cell populations, including monocytes and macrophages.^36^ Because LRRK2 has been implicated in both genetic and sporadic forms of PD, cells lacking LRRK2 or carrying mutant forms of LRRK2 are excellent models to investigate overall mechanisms of PD.^37–39^ In addition to PD, LRRK2 has been implicated in increased risk for stroke, traumatic brain injury, neurocognitive disorders, and depression.^40–44^ While LRRK2 has been well studied in the context of neurons, less is known about the function of LRRK2 in immune cell populations. Previously, we found that loss of LRRK2 in peripheral macrophages including BMDMs, peritoneal macrophages, and macrophage cell lines, results in significantly elevated basal levels of type I IFN/ISGs and an inability to induce interferon responses after infection.^45^ We linked these elevated basal I IFN responses to mitochondrial stress, namely mitochondrial fragmentation and oxidative stress, that causes mitochondrial DNA leakage into the cytosol and chronic engagement of the cGAS/STING signaling pathway.^45^

Here, motivated by our *Lrrk2* KO macrophage findings, we investigated how loss of LRRK2 impacts CNS resident microglia cells. Surprisingly, we found that microglial cells lacking LRRK2 had a reduction in type I IFN transcripts compared to controls. Differential gene expression analysis uncovered that the NRF2 redox pathway was upregulated in *Lrrk2* KO microglia. Consistent with enhanced protection, *Lrrk2* KO microglial cells were better at maintaining a healthy mitochondrial membrane potential and had a higher capacity for OXPHOS, which was dependent on NRF2. Inhibition of LRRK2 kinase activity was sufficient to upregulate NRF2 and reduce ISGs in microglial cells. Surprisingly, LRRK2 kinase inhibition had the opposite effect on peripheral macrophages and reduced NRF2 protein expression. Likewise, macrophages harboring a point mutation that increases LRRK2 kinase activity (*Lrrk2* G2019S) had elevated nuclear NRF2 expression, whereas *Lrrk2* G2019S microglia had reduced NRF2 expression and elevated ISGs. Taken together, these data show LRRK2 kinase function plays differential and active roles in regulating both the type I IFN response and NRF2 activity in microglial cells and macrophages. This work gives insight into the functional requirements of different macrophage populations and how anti- and pro- inflammatory processes are regulated in various tissues.

## Materials and methods

### Mice

*Lrrk2* KO mice (C57BL/6-Lrrk2tm1.1Mjff/J) stock no. 016121, and *Lrrk2* G2019S KI mice (B6.Cg-Lrrk2tm1.1Hlme/J) stock no, 030961 were purchased from The Jackson Laboratories (Bar Harbor, Maine). All mice used in experiments were compared to age- and sex- matched controls by pooling equal males and females between genotypes. To ensure littermate controls were used in all experiments *Lrrk2* KO crosses were made with (KO) *Lrrk2^−/−^* × (HET) *Lrrk2^+/−^* mice. Mice used to make glial cultures were between P1 and P1.5 d old. All animals were housed, bred, and studied at Texas A&M Health Science Center under approved Institutional Care and Use Committee guidelines.

### Primary cells

Mixed glial cultures were differentiated from the brains of neonatal mice as described.^46^ Briefly, glial cells were isolated from the cortexes of neonate mice at P1 to P1.5. Disaggregation media was used to liberate glial cells. Glial cells were centrifuged twice 400 rcf, 5 min and washed in complete media (DMEM, 10% FBS, 1 mM sodium pyruvate, 10% MCSF conditioned media), and grown in 10 mL of media 10 cm TC-treated dishes, 1 dish per brain at 37 °C 5% CO_2_. Complete media was replaced on day 1 following gentle aspiration. Cells were allowed to differentiate in complete media feeding 5 ml on day 5 and then replacing 5 ml of media on every other day afterward. Following 10 d of culture, microglial cells were isolated from glial cultures by washing briefly with cold 1× PBS + EDTA to detach the microglial layer. Cells were then counted, plated on non-tissue culture treated plates, and washed after 4 h with 1× PBS to remove contaminating cell populations. Bone marrow derived macrophages (BMDMs) were differentiated from BM cells isolated by washing mouse femurs with 10 ml DMEM 1 mM sodium pyruvate. Cells were then centrifuged for 5 min at 400 rcf and resuspended in BMDM media (DMEM, 20% FBS [Millipore], 1 mM sodium pyruvate [Lonza], 10% MCSF conditioned media [Watson lab]). BM cells were counted and plated at 5 × 10^6^ cells per 15 cm non-TC treated dishes in 30 mL complete BMDM media. Cells were fed with an additional 15 ml of BMDM media on day 3. BMDMs were harvested on day 7 of culture by washing cells with 1× PBS EDTA (Lonza).

### Cell lines

SIMA9 cells (ATCC^®^ SC-6004™), and RAW 264.7 cells (ATCC^®^ TIB-71™) were obtained from the ATCC. BV2 cells were gifted by Dr Jianrong Li, Texas A&M. For BV2 and SIMA9 cells stably expressing scramble knockdown (KD) and *Lrrk2* KD, Lenti-X cells were transfected with a pSICOR scramble non-targeting shRNA construct and pSICOR *Lrrk2* targeting constructs using Polyjet (SignaGen Laboratories). Virus was collected 24 and 48 h post transfection. Microglia were transduced using Lipofectamine 2000 (Thermo Fisher). After 48 h, the media was supplemented with puromycin (Invitrogen) to select cells containing the shRNA plasmid.

### Flow cytometry

To confirm purity, isolated astrocytes were gated by SSC/FSC and identified as GFAP+. Microglial cells isolated from glial cultures were gated on SSC/FSC followed by CD45+ and defined as CD45+ (eBiosciences) CD11b+ (eBiosciences). Activation markers IAb (eBioceiences), and CD86 (eBiosciences) were analyzed on this population. To assess mitochondrial membrane potential, cells were released from culture plates with 1x PBS + EDTA. Single cell suspensions were made in 1× PBS 2% FBS. For TMRE assays, cells were stained for 20 min at 37 °C in 25 nM TMRE dye and analyzed on an LSR Fortessa X20 (BD Biosciences). Fluorescence was measured under PE (585/15). To assess mitochondrial membrane potential under stress, cells were treated for 15 min with 50 µM FCCP. For JC-1 assays, JC-1 dye was sonicated for 5 min with 30 s intervals. Cells were stained for 20 min at 37 °C in 1 µM JC-1 dye and analyzed on an LSR Fortessa X20 (BD Biosciences). Aggregates were measured under Texas Red (610/20 600 LP) and monomers under FITC (525/50 505 LP). To assess mitochondrial membrane potential under stress, cells were treated for 3 h. with 2.5 µM rotenone prior to being lifted of the culture plates. Also, 5 µM ATP was then added for 5, 15, or 30 min, or 50 µM FCCP was added for 15 min.

### Western blot

Cells were lysed in 1× RIPA buffer with protease and phosphatase inhibitors (Pierce). DNA was degraded using 1 U/ml benzonase (EMD Millipore). Proteins were separated by SDS- PAGE and transferred to nitrocellulose membranes. Membranes were blocked overnight in either 5% BSA or non-fat milk, and incubated overnight at 4 °C with the following antibodies: IBA1 (Wako Chemical 019-19741) 1:2000; RSAD2 (Proteintech) 1:1000; STAT1 (Cell Signaling) 1:1000 pRAB10 T73 (Abcam) 1:1000; IFIT1 (Proteintech) 1:1000; IFIT3 (Proteintech) 1:1000; NRF2 (Cell Signaling), 1:1000; HMOX1 (Proteintech); ACTB (Abcam), 1:5000; and TUBB (Abcam), 1:5000. Membranes were incubated with appropriate secondary antibody (Licor) for 2 h at RT prior to imaging on Odyssey Fc Dual-Mode Imaging System (Licor).

### Immunofluorescence microscopy

Microglia or macrophages were seeded at 2.5 × 10^5^ cells/well on glass coverslips in 24-well dishes. Cells were fixed in 4% paraformaldehyde for 10 min at RT and then washed 3 times with PBS. Coverslips were incubated in diluted primary antibody 1:200 ACTIN (Abcam), 1:200 NRF2 (1:200), in 1× PBS + 5% non-fat milk + 0.3% Triton-X (PBS-MT) for 3 h. Cells were then washed 3 times in 1× PBS and incubated in secondary antibodies at 1:500 for 1 h and then DAPI diluted in PBS-MT for 5 min. Coverslips were washed twice with 1x PBS and twice with deionized water and mounted on glass slides using Prolong Gold Antifade Reagent (Invitrogen),

### Seahorse metabolic assays

Seahorse XF Mito Stress test kits and cartridges (Agilent) were prepared per manufacturer’s protocol and as previously described.^47^ Microglia were seeded at 5 × 10^4^ cells/well and analyzed the following day on a Seahorse XF 96well Analyzer (Agilent). For treatments cells were stimulated overnight with 10 ng/ml LPS (Invivogen), or 100 IU IFN-β (PBL), or for 4 h with 5 µM ML385 (Selleckchem) or 5 µM Brusatol (Selleckchem) for NRF2 inhibition.

### mRNA sequencing

Microglial cell library preparation was carried out by the Baylor College of Medicine Genomic and RNA Profiling Core (GARP) in biological triplicate. RNA sequencing (150 bp paired-end reads) was performed on an Illumina NovaSeq 6000 with S4 flow cell. Data was analyzed by ROSALIND^®^ (https://rosalind.bio/), with a HyperScale architecture developed by ROSALIND, Inc. (San Diego, California). Quality scores were assessed using FastQC. Reads were aligned to the *Mus musculus* genome build GRCm39 using Agilent software. Differentially expressed genes were selected as those with *P*-value threshold <0.05. Transcriptome analysis was performed using IPA analysis to generate GO terms, disease pathway lists, and to compare loss of LRRK2 between microglia and macrophages. Heatmaps were generated using GraphPad Prism software (GraphPad, San Diego, California). Rosalind and IPA were used for pathways analysis and to generate volcano plots and Venn diagrams.

### qRT-PCR

RNA was isolated using Directzol RNAeasy kits (Zymogen). cDNA was made with iScript Direct Synthesis kits (BioRad) per manufacturer’s protocol. Quantitative reverse transcription polymerase chain reaction (qRT-PCR) was performed in triplicate using Sybr Green Power up (ThermoFisher). Data were analyzed on a ViiA 7 Real-Time PCR System (Applied Biosystems) or a BioRad CFX96 analyzer (BioRad).

### Statistical analysis

All data are representative of 3 or more independent experiments with an n = 3 or more. Graphs were generated using Prism (GraphPad). Image analysis was performed using either Image J or cellpose. Significance for assays were determined using a Student 2-tailed *t*-test, or a 1-way ANOVA followed by a Sidak’s multiple comparisons test for more than 2 variables, unless otherwise noted.

## RESULTS

### The type I IFN signature is reduced in microglial cells upon loss of LRRK2.

**Figure 1.**
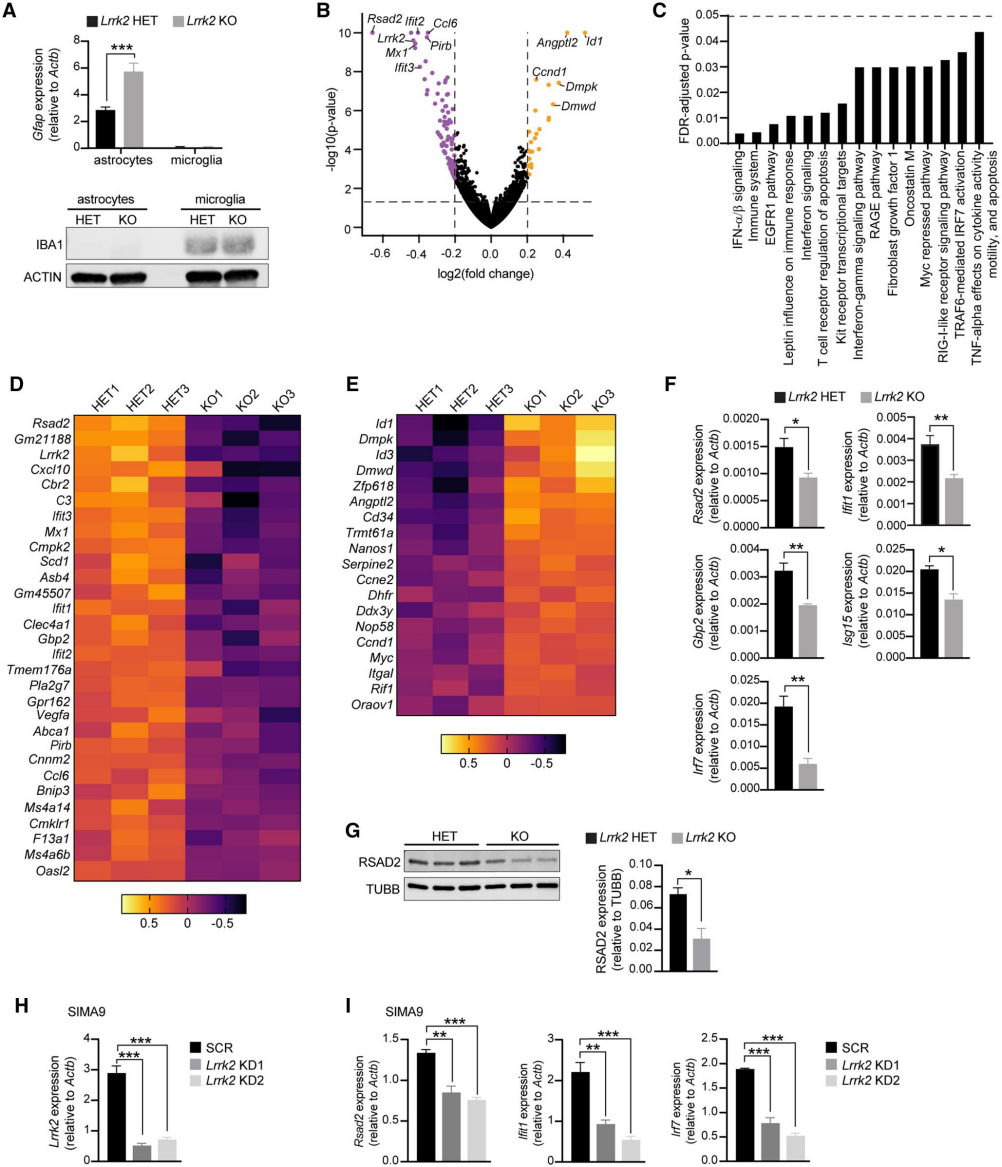
Loss of LRRK2 reduces tonic IFN signaling in microglial cells. (A) Transcript levels of *Gfap* in *Lrrk2* KO and *Lrrk2* HET (control) astrocytes and microglia measured by qRT-PCR (upper graph). Protein levels of IBA1 relative to ACTIN in *Lrrk2* KO and HET astrocytes and microglia measured by western blot (lower graph). (B) Volcano plot of genes differentially expressed between *Lrrk2* KO and HET microglia (left, purple) down in KO, (right, orange) up in KO. (C) Ingenuity pathway analysis of major transcriptional pathways differentially expressed between *Lrrk2* KO and HET microglia (D) Heatmap of significant genes downregulated in *Lrrk2* KO microglia compared to HET controls. (E) Heatmap of significant genes upregulated in *Lrrk2* KO microglia compared to HET controls. (F) Transcript levels of ISGs *Rsad2*, *Ifit1*, *Gbp2*, *Isg15*, and *Irf7* in *Lrrk2* KO and HET microglia measured by qRT-PCR. (G) Protein levels of RSAD2, compared to TUBB in *Lrrk2* KO and HET microglia measured by western blot; n = 3. Quantification on right. (H) Transcript levels *Lrrk2* in *Lrrk2* KD and SCR SIMA9 microglia measured by qRT-PCR. (I) The same as in (H), but ISGs *Rsad2*, *Ifit1*, and *Irf7*. Two-tailed Student *t*-test was used to determine statistical significance. *P < 0.05, **P < 0.01, ***P < 0.005.

To understand how loss of LRRK2 impacts microglial cells, we first developed a process to generate and isolate pure populations of non-activated primary microglial cells from the cerebral cortices of P1.0-P1.5 neonates using sex and age matched littermate controls (*Lrrk2* KO vs. *Lrrk2* HET). Key to this approach is the ability to separate microglial cells from astrocytes, another abundant glial cell population in the brain. We achieved this by dissecting out cerebral cortices followed by a high trypsin digestion (2.5%) to disrupt cells of the meninges followed by 6 days of culture for astrocytes, or 10 d of culture for microglial cells in complete media containing MCSF. Microglial cells were gently washed off the astrocyte layer with PBS/EDTA. We then verified microglial vs. astrocyte cell populations measuring GFAP mRNA expression (an abundant astrocyte transcript) and IBA1 protein (a key surface marker of microglia) (Fig. 1A). Astrocyte purity was also assessed by measuring GFAP+ cells (>90%) by flow cytometry (Fig. S1A). Microglia, defined as CD45+ CD11b+, were measured to be >85% pure by flow cytometry (Fig. S1B). To identify the major LRRK2-dependent differences in transcript abundance, we performed bulk RNA-SEQ analysis from RNA collected from resting microglia and generated sequencing libraries. Using the Rosalind RNA-seq platform, we compared transcripts between HET and *Lrrk2* KO microglia and identified 96 significantly differentially expressed genes (adj. *P*-value < 0.05), with 77 downregulated and 19 upregulated genes (Fig. 1B, Fig. S1B, Table S1). Notably, many of the most significantly down regulated genes, aside from *Lrrk2*, were ISGs including *Rsad2*, *Ifit2*, *Mx1*, *Ifit3*, *Cxcl10*, *Cmpk2*, among others (Fig. 1B, D purple). Consistent with the type I IFN pathway being impacted, pathway analysis through NCATS BioPlanet identified the most significant pathway impacted as IFN α/β signaling (Fig. 1C). Only a handful of genes were significantly upregulated in *Lrrk2* KO microglia. These genes (*Id1*, *Id3*, *Dmpk*, *Cd34*, *Serpine2 and Dhfr*) have been associated with neurogenesis and microglial cell division^48–52^ (Fig. 1E). qRT-PCR confirmed downregulation of multiple ISG transcripts including but not limited to *Rsad2*, *Ifit1*, *Gbp2*, *Isg15*, and *Irf7* (Fig. 1F). Importantly, downregulation of ISGs was also observed at the protein level (Fig. 1G, Fig. S1D). By generating lentiviral shRNA knockdowns (KDs) of *Lrrk2* alongside a scramble (SCR) control, we observed the same reduced type I IFN signature in the spontaneously immortalized microglial cell line SIMA9 (Fig. 1H, I), supporting a role for LRRK2 in promoting ISG expression in microglial cells.

To better understand potential drivers of this phenotype, we looked toward known regulators of the type I IFN response in macrophages.^53^^,^^54^ We saw no major differences in activation (I-Ab) or costimulatory marker (CD86) expression, indicating that loss of LRRK2 did not impact major states of cellular activation (Fig. S1E). Likewise, we saw no difference in the expression of negative regulators of type I IFN gene expression like *Sosc1*, *Smad2/3*, and *Pias4* (Fig. S1F).^55^ We also confirmed that genes associated with M1-like vs M2-like macrophage states were not differentially regulated in *Lrrk2* KO microglia (the type I IFN response has previously been linked to M1 polarization through *Irf7*^56^ (Fig. S1G). Taken together, these data argue that LRRK2 is required to maintain tonic levels of ISGs in microglial cells through a previously undescribed mechanism.

### Loss of LRRK2 differentially impacts microglia and peripheral macrophages.

**Figure 2.**
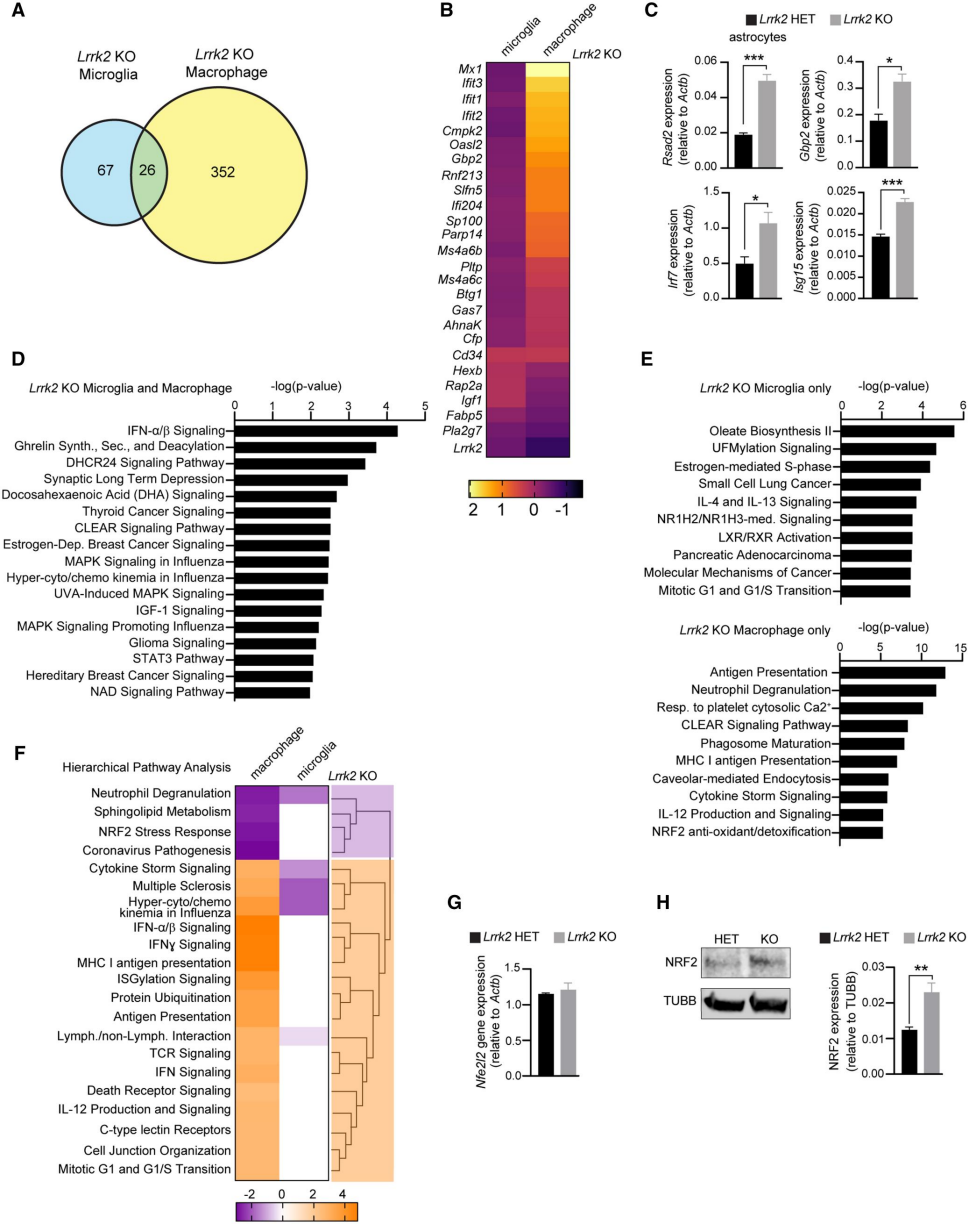
The type I IFN response and NRF2 pathways are opposed in microglial cells and macrophages. (A) Venn diagram depicting genes differentially expressed in *Lrrk2* KO microglial cells (left), both *Lrrk2* KO microglia and macrophages (center) or only *Lrrk2* KO macrophages (right). (B) Heatmap of genes differentially expressed in *Lrrk2* KO microglia and macrophages. (C) Transcript levels of ISGs *Rsad2*, *Gbp2*, *Irf7*, *Isg15*, in *Lrrk2* KO and HET astrocytes measured by qRT-PCR. (D) IPA pathway plot of shared pathways impacted by a loss of LRRK2 in microglia and macrophages. (E) Pathway analysis of genes upregulated in *Lrrk2* KO microglia only (upper) and Lrrk2 KO macrophages only (lower). (F) Hierarchal clustered heatmap depicting z-scores and pathway differences between microglial cells and macrophages. (G) Transcript levels of *Nfe2l2* (NRF2) in *Lrrk2* KO and HET microglia measured by qRT-PCR (H) The same as in (G) but NRF2 proteins levels relative to TUBB. Two-tailed Student *t*-test was used to determine statistical significance. *P < 0.05, **P < 0.01, ***P < 0.005.

Given the surprising phenotype of *Lrrk2* KO microglia, we decided to compare the transcriptional profile differences of *Lrrk2* KO microglia to *Lrrk2* KO peripheral macrophages. We observed an overlap of 26 genes differentially expressed genes (DEGs) in both *Lrrk2* KO microglia and macrophages, with 67 and 352 DEGs distinct for microglia and macrophages, respectively (Fig. 2A, Tables S1, S2). Within the group of 26 shared DEGs, many genes were oppositely impacted by loss of LRRK2 in macrophages and microglial cells, with 19 genes increased in macrophages but reduced in microglia (Fig. 2B). Half of these “conflicting” DEGs, including *Mx1*, *Ifit1-3*, *Oasl2*, and *Gbp2,* are associated with the type I IFN response and Ingenuity Pathway Analysis identified “IFN-α/β Signaling” as the most significantly impacted pathway shared between *Lrrk2* KO macrophages and microglia (Fig. 2D).

We initially hypothesized that the type I IFN phenotype in *Lrrk2* KO microglia could have something to do with the CNS residency of these cells. To test if other CNS glial cells also downregulated type I IFN in the absence of LRRK2, we performed qRT-PCR analysis on *Lrrk2* KO and HET astrocytes, purified as described in (Fig. 1A, Fig. S1A). Unexpectedly, we found that ISGs were upregulated in *Lrrk2* KO astrocytes compared to HET controls (Fig. 2C). We also noted that transcripts associated with astrocyte maturation and activation, for example, *Gfap*, *S100b*, *Icam1*, and *Ccl5,* were elevated in *Lrrk2* KO astrocytes^57^ (Fig. S2). These data suggest that *Lrrk2* KO astrocytes display a more macrophage-like type I IFN phenotype and that the downregulation of ISGs in *Lrrk2* KO microglia is not shared by other CNS-resident glial populations.

To better understand why loss of LRRK2 differentially impacts ISG expression in astrocytes/macrophages vs. microglia, and perhaps identify the driver of this phenotype, we performed pathway analysis of non-type I IFN genes in *Lrrk2* KO microglia and macrophages. We found that non-ISG DEGs in *Lrrk2* KO microglial cells were enriched in pathways related to cell cycle, metabolism, and cancer (Fig. 2E upper graph), whereas non-ISG DEGs in *Lrrk2* KO macrophages were enriched in pathways related to immune-mediated processes, including antigen presentation, neutrophil degranulation, cytokine signaling, and NRF2-mediated antioxidant protection (Fig. 2E lower graph). To begin to understand what differentially regulated pathways might be contributing to the inverse phenotype of *Lrrk2* KO macrophages vs. microglia, we performed hierarchical clustering of the most significant differentially regulated pathways. (Fig. 2F). Two major clusters were noted. One cluster contained upregulated inflammatory signaling pathways (Fig. 2F, orange box); the other contained several pathways downregulated only in *Lrrk2* KO macrophages, including sphingolipid metabolism, NRF2 stress response, and pathogenesis of coronavirus (Fig. 2F, purple box). We chose to focus on NRF2 due to its previous association with downregulating type I IFN responses.^23^^,^^25^^,^^32^

To establish that NRF2 expression could be impacted by loss of LRRK2, we performed qRT-PCR (Fig. 2G), and western blot analysis (Fig. 2H) of NRF2 in *Lrrk2* KO and HET microglia. We found that NRF2 expression was increased at the protein but not mRNA levels, suggesting that NRF2 redox sensing is upregulated in the absence of LRRK2. Taken together, these data argue that compared to peripheral macrophages, microglial cells employ additional regulatory nodes to restrict type I IFN responses that rely on the redox regulator NRF2.

### *Lrrk2* KO microglia are protected from stressors and have enhanced mitochondrial metabolism during activation.

**Figure 3.**
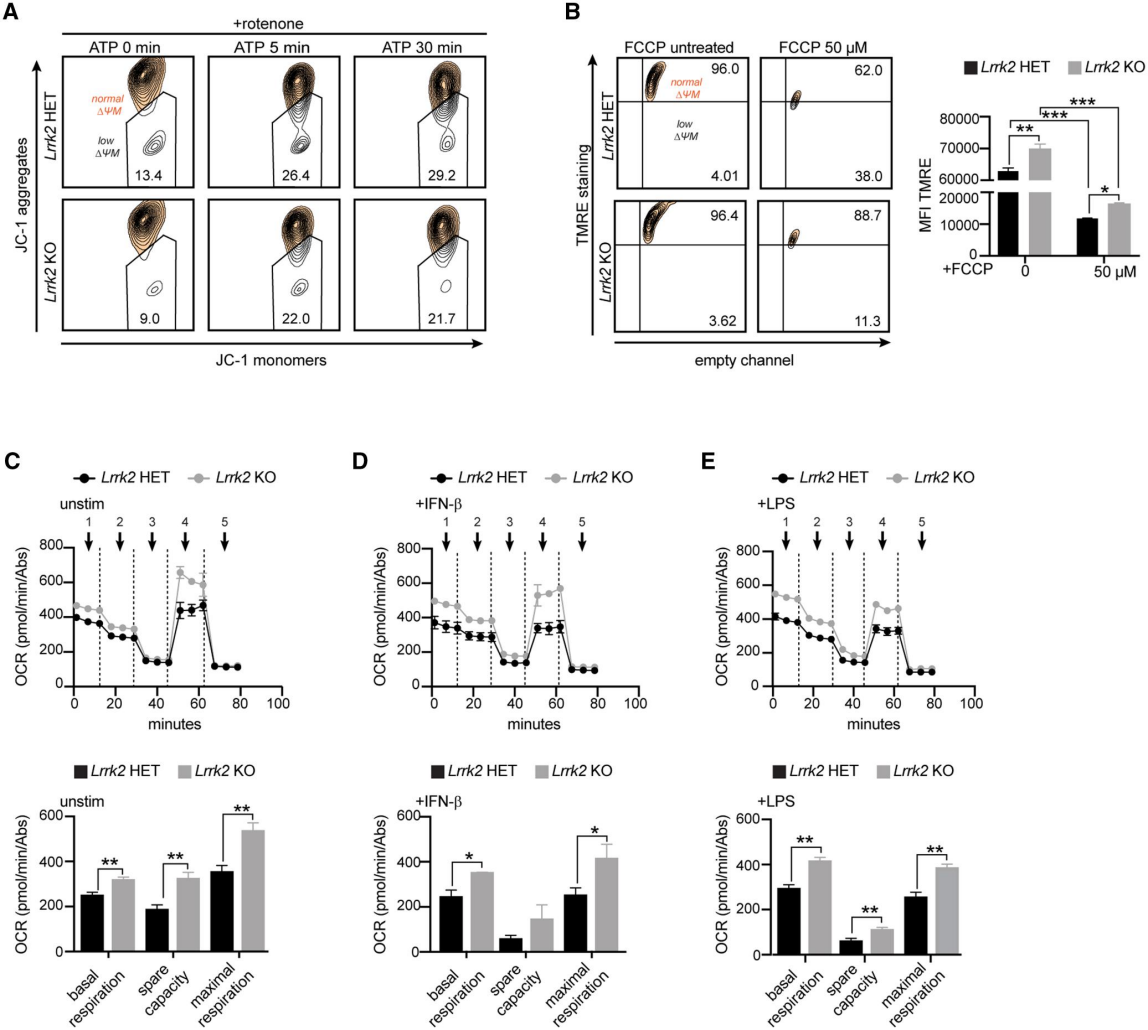
Loss of LRRK2 promotes mitochondrial protection in microglial cells. (A) JC-1 staining of mitochondrial membrane potential in *Lrrk2* KO and HET microglia measured by flow cytometry. Cells were treated with 2.5 µM rotenone for 3 h. followed by 5 µM ATP for 5 and 30 min. (B) TMRE staining of mitochondrial membrane potential in *Lrrk2* KO and HET microglia measured by flow cytometry. Cells were treated with vehicle control (untreated) or 50 µM FCCP for 30 min. (C) Oxygen consumption rate (OCR) of resting *Lrrk2* KO and HET microglia measured by the Seahorse analyzer mito-stress test. Arrows and numbers indicate reads between injections times. Quantification of major OCR readouts below. (D) The same as in (C) but cells were treated for 16 h with 100 IU IFN-β. (E) The same as in (C) and (D) but cells were treated for 16 h with 10 ng/ml LPS. Two-tailed Student *t*-test or 1-way ANOVA with Sidak’s multiple comparisons was used to determine statistical significance. *P < 0.05, **P < 0.01, ***P < 0.005.

Because we previously found that the increase in LRRK2-dependent basal type I IFN in macrophages was linked to mitochondrial dysfunction, including reduced mitochondrial membrane potential and decreased OXPHOS,^45^ we hypothesized that *Lrrk2* KO microglial mitochondria would have the inverse phenotype. To test this, we performed mitochondrial membrane potential assays using JC-1 and TMRE on *Lrrk2* KO and HET microglia. JC-1 is a carbocyanine dye that accumulates within healthy mitochondria with normal membrane potential to form red fluorescent aggregates. Upon loss of mitochondrial membrane potential, JC-1 diffuses to the cytosol as a monomer where it emits a green fluorescence, providing a facile tool to determine mitochondrial health. In line with our hypothesis, loss of LRRK2 resulted in increased mitochondrial membrane potential (more red aggregates) in resting microglia and microglia exposed to rotenone/ATP (Fig. 3A). We further confirmed this by using the mitochondrial membrane potential dye TMRE in resting *Lrrk2* KO and HET microglia (Fig. S3A, Fig. 3B), and microglia treated with the uncoupling agent FCCP (Fig. 3B). Consistent with an inverse correlation between type I IFN upregulation and mitochondrial health, we saw the opposite response in astrocytes (Fig. S3C). Protection of mitochondrial membrane potential was also observed in *Lrrk2* KD SIMA9 microglial cells compared to SCR control (Fig. S3B). Given that *Lrrk2* KO microglial mitochondria had increased membrane potential even under high stress conditions, we next used the Agilent Seahorse Metabolic Analyzer to further investigate the impact of stress on the mitochondrial metabolic state. In the Seahorse assay, oxidative phosphorylation (OXPHOS) and glycolysis are assayed by oxygen consumption rate (OCR) and extracellular acidification rate (ECAR), respectively. We found that OCR in *Lrrk2* KO microglia was enhanced in terms of basal, spare and maximal capacity (Fig. 3C, lower quantification), indicating that *Lrrk2* KO mitochondria were not only more active at rest, but they had a greater capacity for maintaining high levels of OXPHOS during stress, indictive of a protected state. This protection was maintained following activation with type I IFN, which can enhance OCR in macrophages^58^ (Fig. 3D, lower quantification), as well as LPS, which has been shown to reduce OCR in macrophages^59^ (Fig. 3E, lower quantification). While oxidative phosphorylation relied heavily on LRRK2, glycolysis, as measured by ECAR, was not impacted under any of these conditions (Fig. S3C–E). These data are consistent with enhanced protection of mitochondria in *Lrrk2* KO microglia and suggest that LRRK2 negatively regulates NRF2 to restrict OXPHOS capacity in microglia.

### Upregulation of NRF2 drives mitochondrial protection in *Lrrk2* KO microglial cells

**Figure 4.**
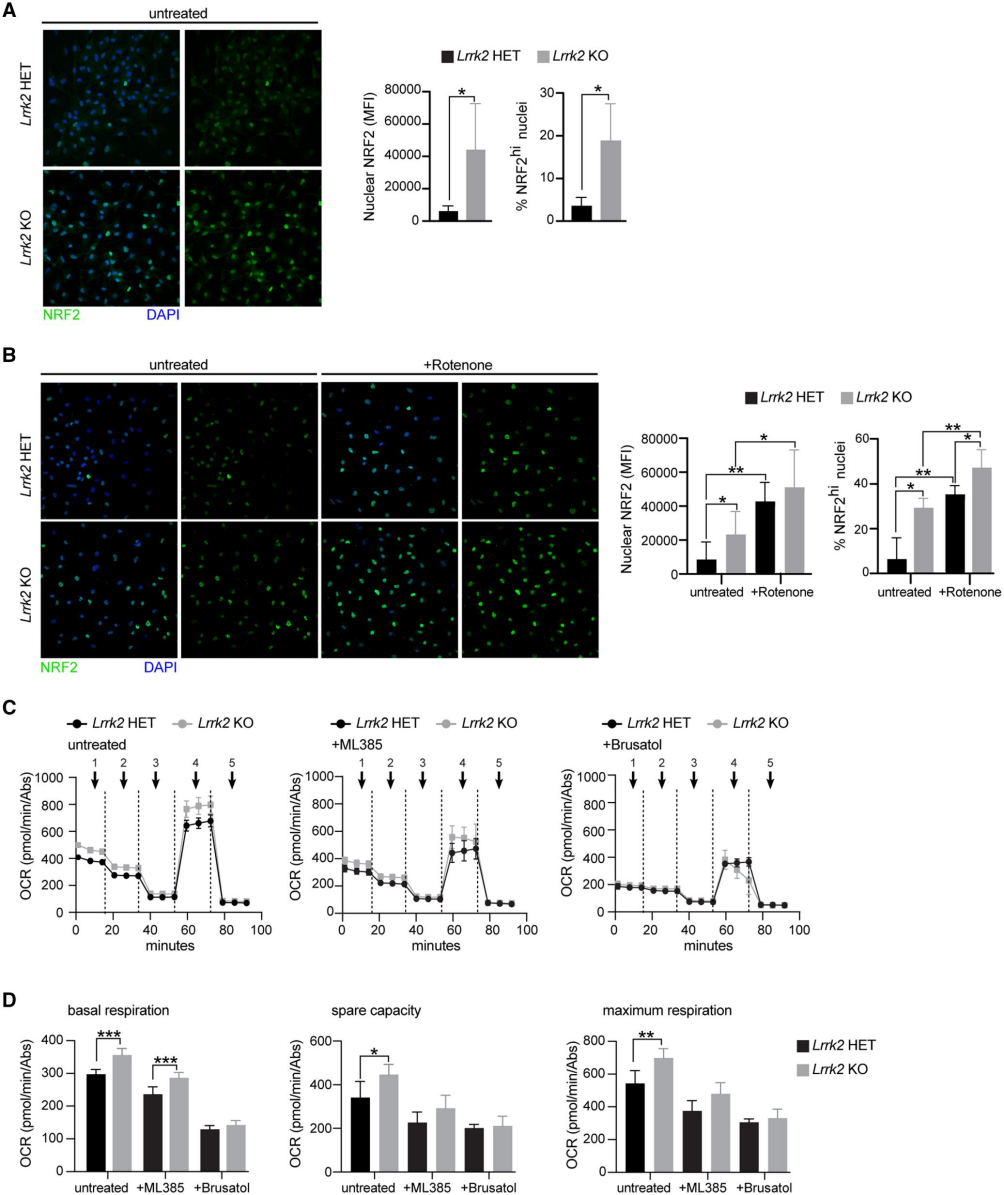
Upregulation of NRF2 drives mitochondrial protection in LRRK2 KO microglial cells. (A) Protein levels and localization of NRF2 in resting *Lrrk2* KO and HET microglia measured by Immunofluorescence microscopy NRF2 (green), DAPI (blue). Left graph measures NRF2 expression based on mean fluorescence intensity, right graph measures the number of NRF2^hi^ nuclei per field of vision (B) The same as in (A) but cells were treated with 200 ng/mL rotenone for either 0 or 4 hrs. to induce mitochondrial stress. (C) Oxygen consumption rate (OCR), a proxy for oxidative phosphorylation, of *Lrrk2* KO and HET microglia measured by the Seahorse analyzer mito-stress test. Cells were either treated with vehicle control (untreated) (left panel) or treated with NRF2 inhibitors for 4 hrs., ML385 5 µM (middle panel), Brustol 5 µM (right panel). (D) Quantification of the major OCR readouts from the above line graphs. Two-tailed Student *t*-test or 1-way ANOVA with Sidak’s multiple comparisons was used to determine statistical significance. *P < 0.05, **P < 0.01, ***P < 0.005.

In addition to downregulating the type I IFN response directly, NRF2 has been also shown to protect the mitochondria through the upregulation of reactive oxygen scavengers, induction of autophagy, and other protective metabolic programs.^60–63^ We therefore choose to further explore the divergence between NRF2 in microglial cells and macrophages focusing on NRF2 protective capacity and the mitochondria. To begin to understand how NRF2 might be differentially regulated in *Lrrk2* KO microglial cells compared to controls, we examined both NRF2 expression and localization. Cytoplasmic NRF2 is bound to the KEAP1 complex where it is ubiquitinated and constitutively degraded by the proteosome.^64^ During cellular stress, KEAP1 releases NRF2, allowing it to rapidly translocate to the nucleus and turn on protective gene expression programs. Consistent with a more activated (protective) state in *Lrrk2* KO microglia, we observed elevated levels of nuclear NRF2 (green) colocalizing with DAPI (blue) (Fig. 4A) and an increase in the overall expression of NRF2. Consistent with enhanced protection we also saw greater induction of nuclear NRF2 in *Lrrk2* KO microglia treated with rotenone for 4 h to induce mitochondrial stress (Fig. 4B, right graph). Because *Lrrk2* KO microglia showed enhanced mitochondrial OXPHOS at baseline and during stress with greater OXPHOS reserves, we next wanted to ask if this protection was dependent on NRF2. We therefore performed the Seahorse Mito Stress test on *Lrrk2* KO and HET microglia in the presence or absence of NRF2 inhibitors ML385 and brusatol, which inhibit NRF2’s DNA binding ability and expression, respectively.^65^^,^^66^ Compared to untreated *Lrrk2* KO microglia (Fig. 4C, left panel), we found that *Lrrk2* KO microglia treated with ML385 (Fig. 4C, central panel) or brusatol (Fig. 4C right panel), lost their protective metabolic state and exhibited reduced basal, spare and maximal respiration potential compared to controls (Fig. 4D). Taken together, these data indicate that NRF2 is elevated and activated in *Lrrk2* KO microglia compared to HETs, and this enhanced activity provides protection to the mitochondria.

### LRRK2 kinase activity promotes tonic ISG expression and tempers NRF2 activity

**Figure 5.**
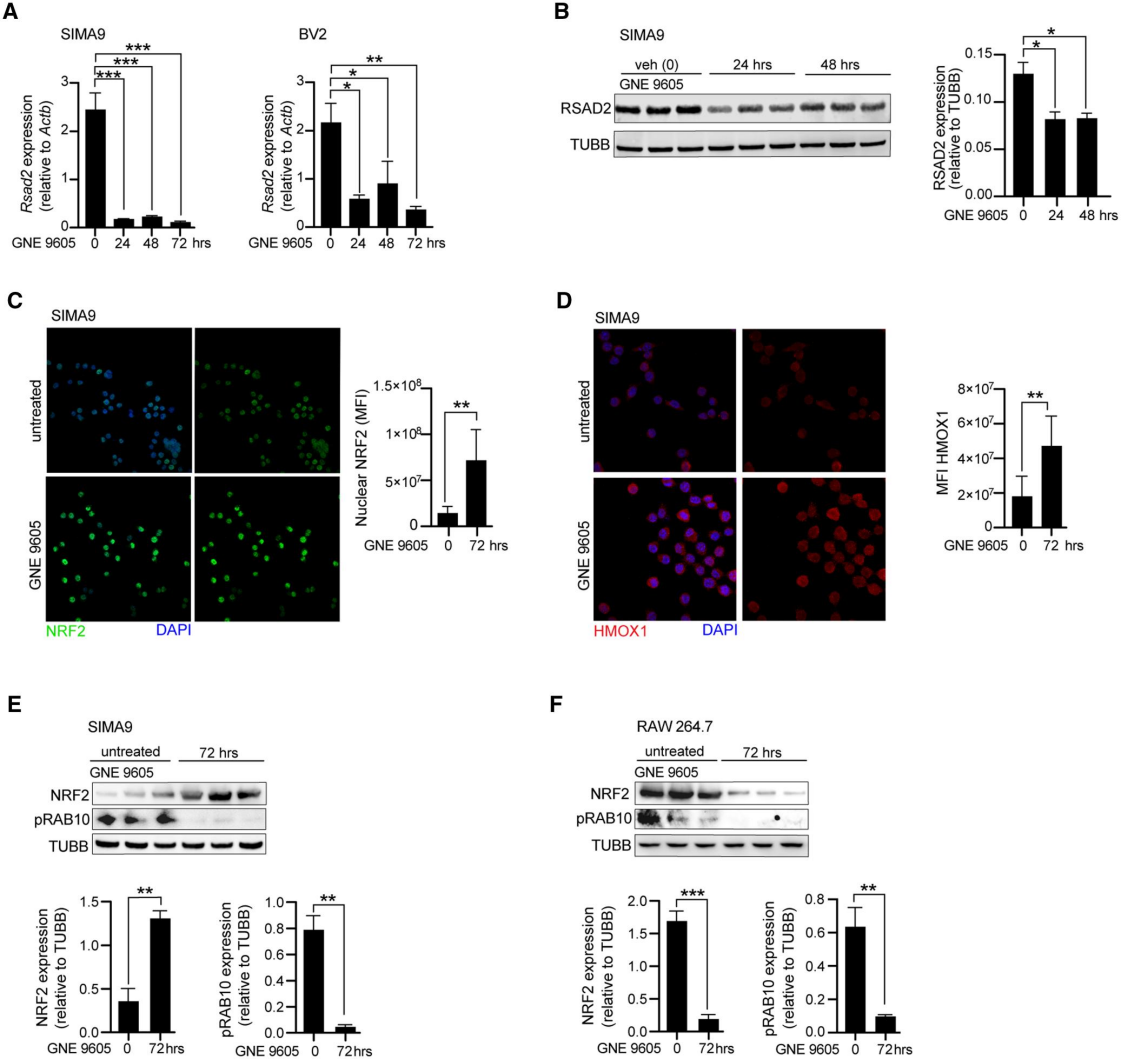
LRRK2 kinase activity regulates the type I IFN response and NRF2 activity in microglial cells. (A) Transcript levels of *Rsad2*, in SIMA9 and BV2 microglia measured by qRT-PCR. Cells were treated for 0, 24, 48, and 72 h. with the small molecule LRRK2 inhibitor 10 µM GNE9605. (B) Protein levels of RSAD2, compared to TUBB in SIMA9 microglia measured by western blot. Cells were treated for 24 and 48 h. with 10 µM GNE9605 (C) Protein levels and localization of NRF2 in SIMA9 microglia treated with or without 10 µM GNE9605 for 72 h. measured by immunofluorescence microscopy NRF2 (green), DAPI (blue). (D) The same as in (C) but looking at HMOX1 (red). (E) Protein levels as measured by western blot for NRF2 and pRAB10 T73 in SIMA9 cells treated for 72 h with 10 µM GNE9605 or vehicle control (untreated). Protein normalized to tubulin (TUBB). Quantification on right. (F) The same as in (E) but using RAW 264.7 cells. Two-tailed Student *t*-test or 1-way ANOVA with Sidak’s multiple comparisons was used to determine statistical significance. *P < 0.05, **P < 0.01, ***P < 0.005.

Because increased LRRK2 kinase activity is associated with pathophysiology of both familial and sporadic PD,^38^^,^^67^^,^^68^ inhibition of LRRK2 kinase activity has been proposed as a possible route of PD intervention. Currently multiple LRRK2 kinase inhibitors are being tested in clinical trials for PD therapeutics.^69–71^ Given the importance of the type I IFN response in multiple neurodegenerative disorders, we wanted to determine if LRRK2 kinase activity was necessary for controlling ISG expression and restricting NRF2 activity. To this end, we treated SIMA9 and BV2 cells with the blood brain barrier (BBB) penetrant small molecule inhibitor of LRRK2, GNE9605. We found that as early as 24 h post-treatment with GNE9605, the ISG transcript *Rsad2* was depleted in both microglial cell lines (Fig. 5A) and RSAD2 protein levels were reduced in GNE9605-treated SIMA9 cells (Fig. 5B). The decrease in RSAD2 expression caused by LRRK2 kinase inhibition was dependent on NRF2 activity and expression, as cotreatment with ML385 partially prevented the reduction in RSAD2 (Fig. S4A). LRRK2 kinase activity also restricted the NRF2 antioxidant response, as SIMA9 microglia treated with GNE9605 had elevated nuclear expression of NRF2 (Fig. 5C) and increased expression of the NRF2-induced antioxidant target protein HMOX1 (Fig. 5D). To better understand the differences between the LRRK2 kinase dependent control of NRF2 activity in microglia vs. macrophages, we performed a side-by-side comparison of NRF2 expression in SIMA9 microglial cells compared to the macrophage cell line RAW264.7 in the presence of LRRK2 kinase inhibition. We found that LRRK2 kinase inhibition by GNE9605 significantly increased the expression of NRF2 measured by western blot in SIMA9 cells (Fig. 5E) but reduced expression of NRF2 in RAW 264.7 cells (Fig. 5D). In both cell types, phosphorylation of RAB10 at T73, a known LRRK2 target protein, was used as a control to measure LRRK2 kinase inhibition (Fig. 5E, F). Together, these data further support cell-type specific roles for LRRK2 kinase activity in modulating NRF2.

### The PD-associated *Lrrk2* G2019S mutation divergently regulates NRF2 expression in peripheral macrophages and microglial cells

**Figure 6.**
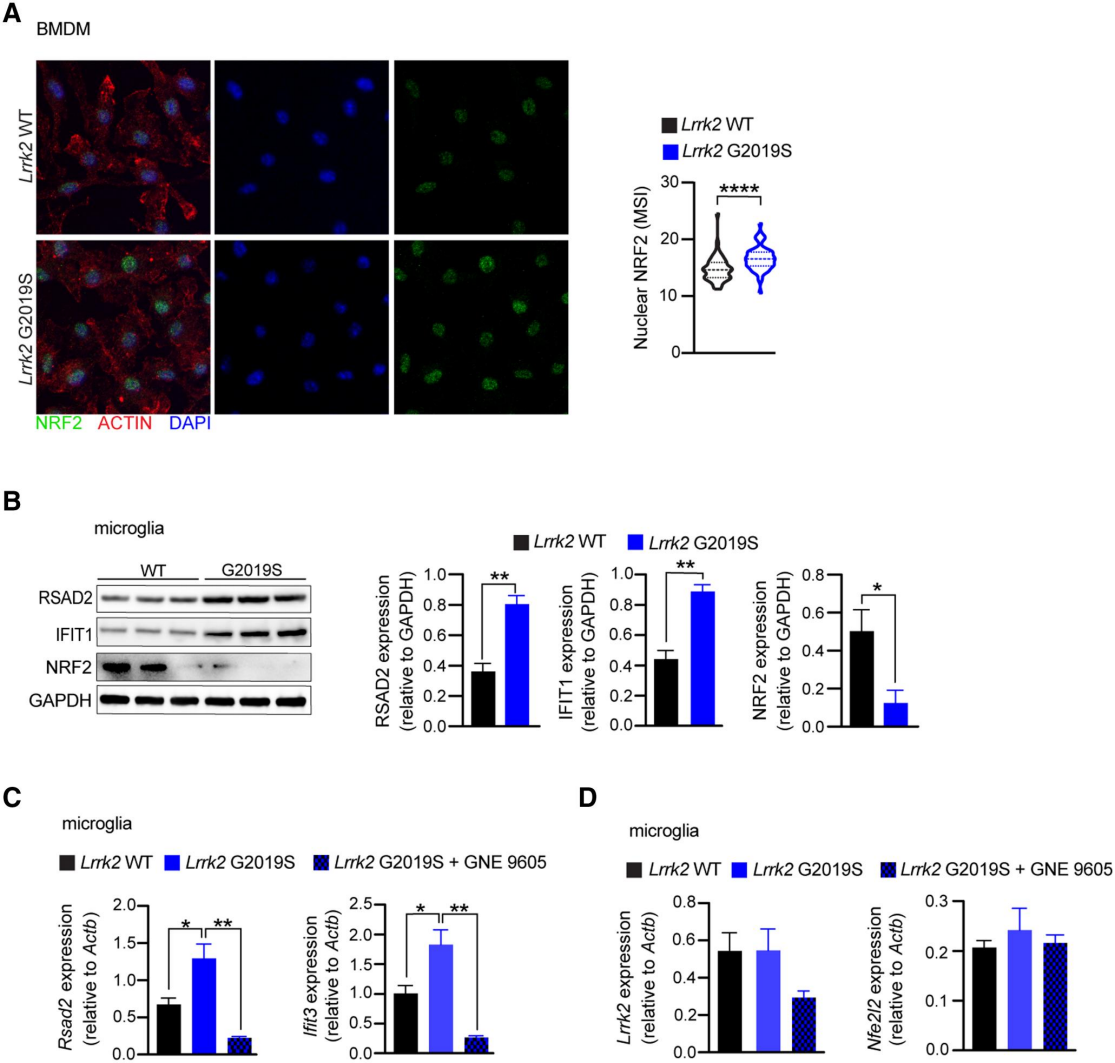
The G2019S mutation in LRRK2 has opposing effects on NRF2 expression in macrophages and microglia. (A) Protein levels and localization of NRF2 in resting *Lrrk2* G2019S and WT BMDMs measured by immunofluorescence microscopy NRF2 (green), DAPI (blue) ACTIN (red). Graph measures NRF2 expression within the nuclei based on mean signal intensity. (B) Protein levels of RSAD2, IFIT1 and NRF2 compared to GAPDH in resting primary microglia from *Lrrk2* G2019S and WT mice measured by western blot. (C) Transcript levels of *Rsad2*, and *Ifit3* measured by qRT-PCR in *Lrrk2* G2019S and WT microglia treated for 24 h with 10 µM GNE9605 or vehicle control. (D) The same as in (C) but looking at *Lrrk2* and *Nfe2l2* expression. Two-tailed Student *t*-test or 1-way ANOVA with Sidak’s multiple comparisons was used to determine statistical significance. *P < 0.05, **P < 0.01, ***P < 0.005.

Given the inverse response LRRK2 kinase inhibition had on NRF2 expression between microglia and macrophages, we lastly wanted to explore the impact of increased LRRK2 kinase activity in peripheral macrophages compared to microglial cells. The PD-associated missense mutation *LRRK2* G2019S has been shown to enhance the kinase activity of LRRK2.^72^^,^^73^ We previously reported that LRRK2 mutant macrophages (*Lrrk2* G2019S) were prone to proinflammatory cell death and fragmented mitochondria.^74^ However, *Lrrk2* G2019S macrophages did not have an elevated tonic ISG signature like *Lrrk2* KO macrophages, which was surprising given the state of their mitochondrial network.^74^ We hypothesized that the increased kinase activity of LRRK2 increased NRF2 levels, contributing to antioxidant protection and dampened type I IFN responses. Consistent with this hypothesis *Lrrk2* G2019S BMDMs had increased nuclear NRF2 expression compared to controls (Fig. 6A).

In contrast to BMDMs, when we looked at NRF2 protein levels in *Lrrk2* G2019S primary microglial cells compared to WT controls we observed reduced NRF2 protein expression and increased ISG proteins including RSAD2 and IFIT1 (Fig. 6B). Elevated ISGs were also visible at the mRNA level and were dependent on LRRK2 kinase activity as treatment with GNE6905 for 24 h was sufficient to reduce expression of ISGs *Rsad2* and *Ifit3* (Fig. 6C). Transcript expression of *Lrrk2* was not impacted by the G2019S mutation nor was expression of *Nfe2l2*, the gene that encodes NRF2 (Fig. 6D). Taken together, these data demonstrate that microglial cells rely on a novel LRRK2-NRF2 circuit to control type I IFN expression that is LRRK2 kinase-dependent. Peripheral macrophages, on the other hand, employ an opposing regulon, providing an example of how different macrophage populations evolve regulatory mechanisms to meet the requirements of their tissue environment.

## Discussion

Despite being most famously associated with antiviral immunity, the type I IFN response is also necessary for the development of healthy neurons, neuron survival, and neurite outgrowth.^17^ Consistent with playing a role in neuronal function, type I IFN is gaining traction as a key mediator of neurodegenerative diseases including Alzheimer’s and PD, in addition to neuroinflammatory conditions like ischemic stroke. It follows then, that resident CNS glial immune cells need ways to regulate expression of type I IFN and ISGs, both in the healthy brain and in response to pathogens or inflammatory triggers. Because little is known about type I IFN regulation in glia, we sought to gain understanding of how PD-related protein, LRRK2, influences immune responses in microglia compared to peripheral macrophages. We found that loss of *Lrrk2* resulted in significantly reduced tonic type I IFN signaling compared to WT or heterozygous (*Lrrk2* HET) controls in both primary microglia and microglial cell lines (Fig. 1). We linked this decrease in basal type I IFN to upregulation of the transcription factor nuclear factor erythroid 2–related factor 2 (NRF2), which promotes cellular resistance to oxidative stress (Fig. 4B, C). Conversely, loss of *Lrrk2* in astrocytes or peripheral macrophages, resulted in an increase in type I IFN, reduced NRF2 expression, and a failure to protect cells from oxidative stress (Fig. 2C, Fig. S2C). Our findings suggest fundamental differences in LRRK2-dependent immune regulation between microglia, other CNS resident immune cells, and the periphery.

The current type I IFN paradigm in macrophages is that cells are primed via cytosolic sensing of mitochondrial DNA through a cGAS/STING dependent axis to maintain tonic type I IFN signaling, allowing for rapid upregulation of IFN-β and ISGs upon viral infection.^75^ The capacity of mitochondrial DNA to prime through cGAS/STING has previously been shown in microglia from aged mice and *Lrrk2* KO peripheral macrophages.^22^ This study suggests that additional regulation of type I IFN is present in microglia from neonatal mice where a loss of LRRK2 is sufficient to downregulate ISGs and protect mitochondria through upregulation of the redox associated transcription factor NRF2. This disparity indicates that in microglial cells, type I IFN priming is either unnecessary or detrimental directly after birth, at time of intense neural development.^76^^,^^77^ These combined findings of microglial type I IFN regulation highlight new and interesting avenues for further research. Future studies investigating the role of LRRK2 in microglia across a broad age range of mice will likely yield exciting insights, considering PD and other neurodegenerative diseases often develop later in life. Given the importance of type I IFN in neuron development, it is understandable that additional regulatory nodes would be necessary to prevent runaway neuroinflammation, which is seen in young mice lacking NRF2.^35^^,^^78–81^ The idea that cGAS/STING primes old but not young microglia is especially intriguing and suggests that NRF2 protection is lost over time, possibly to enhance antiviral immunity in the CNS. This same loss of NRF2 protection could also act as a major factor in the pathobiology of diseases like Alzheimer’s and PD, which have a strong type I IFN component to disease progression.

Previous work investigating PD pathology highlights the importance of NRF2-mediated protection. SNPs in *NFE2L2* that decrease antioxidant function are associated with early onset PD and susceptibility to PD.^82^^,^^83^ Additionally, several drug studies have shown NRF2 activators protect against PD pathology and neurodegeneration in mouse models.^84–87^ In addition to being protective in neurodegenerative disease, activation of NRF2 also ameliorates damage caused from ischemic stroke where it protects the BBB, improves edema, and mitigates neurological defects by reducing oxidative stress.^79^^,^^88^ One mechanism of action is NRF2 restriction of hyperactive type I IFN in microglia which occurs during the acute phase of stroke.^89^ Upregulation of NRF2 could also limit mitochondrial DNA release and subsequent activation of the type I IFN response. LRRK2 kinase inhibitors are also prime targets for use as disease modifying agents in PD, stroke, and traumatic brain injury.^40^^,^^41^^,^^69^ Earlier studies have shown that LRRK2 kinase inhibition upregulates the AMPK/p38 pathway, which is known to regulate NRF2.^90^^,^^91^ Thus, it is possible that LRRK2 control of AMPK signaling could trigger NRF2 upregulation to increase protection against mitochondrial stress and reduce deleterious type I IFN. By defining novel links between LRRK2, NRF2, and mitochondrial homeostasis, our findings can now be further expanded upon in the context of disease-associated *Lrrk2* mutations.

## Supplementary Material

vkaf215_Supplementary_Data

## References

[1] Askew K et al Coupled proliferation and apoptosis maintain the rapid turnover of microglia in the adult brain. Cell Rep. 2017;18:391–405. 28076784 10.1016/j.celrep.2016.12.041 PMC5263237

[2] Kierdorf K et al Microglia emerge from erythromyeloid precursors via Pu.1-and Irf8-dependent pathways. Nat Neurosci. 2013;16:273–280. 23334579 10.1038/nn.3318

[3] Ginhoux F , Lim S, Hoeffel G, Low D, Huber T. Origin and differentiation of microglia. Front Cell Neurosci. 2013;7:45. 23616747 10.3389/fncel.2013.00045 PMC3627983

[4] Ginhoux F et al Fate mapping analysis reveals that adult microglia derive from primitive macrophages. Science. 2010;330:841–845. 20966214 10.1126/science.1194637 PMC3719181

[5] Alliot F , Godin I, Pessac B. Microglia derive from progenitors, originating from the yolk sac, and which proliferate in the brain. Dev Brain Res. 1999;117:145–152. 10567732 10.1016/s0165-3806(99)00113-3

[6] Gomez Perdiguero E et al Tissue-resident macrophages originate from yolk-sac-derived erythro-myeloid progenitors. Nature. 2015;518:547–551. 25470051 10.1038/nature13989 PMC5997177

[7] Paolicelli RC et al Synaptic pruning by microglia is necessary for normal brain development. Science. 2011;333:1456–1458. 21778362 10.1126/science.1202529

[8] Zengeler KE , Lukens JR. Innate immunity at the crossroads of healthy brain maturation and neurodevelopmental disorders. Nat Rev Immunol. 2021;21:454–468. 33479477 10.1038/s41577-020-00487-7 PMC9213174

[9] Salter MW , Stevens B. Microglia emerge as central players in brain disease. Nat Med. 2017;23:1018–1027. 28886007 10.1038/nm.4397

[10] Chien C-H , Lee M-J, Liou H-C, Liou H-H, Fu W-M. Microglia-derived cytokines/chemokines are involved in the enhancement of LPS-induced loss of nigrostriatal dopaminergic neurons in DJ-1 knockout mice. PLoS One. 2016;11:e0151569. 26982707 10.1371/journal.pone.0151569 PMC4794203

[11] Davalos D et al ATP mediates rapid microglial response to local brain injury in vivo. Nat Neurosci. 2005;8:752–758. 15895084 10.1038/nn1472

[12] Cowan MN , Sethi I, Harris TH. Microglia in CNS infections: insights from *Toxoplasma gondii* and other pathogens. Trends Parasitol. 2022;38:217–229. 35039238 10.1016/j.pt.2021.12.004 PMC8852251

[13] McGeer PL , Itagaki S, Akiyama H, McGeer EG. Rate of cell death in Parkinsonism indicates active neuropathological process. Ann Neurol. 1988;24:574–576. 3239957 10.1002/ana.410240415

[14] McGeer PL , Itagaki S, Boyes BE, McGeer EG. Reactive microglia are positive for HLA-DR in the: substantia nigra of Parkinson’s and Alzheimer’s disease brains. Neurology. 1988;38:1285–1291. 3399080 10.1212/wnl.38.8.1285

[15] Bartels T , De Schepper S, Hong S. Microglia modulate neurodegeneration in Alzheimer’s and Parkinson’s diseases. Science. 2020;370:66–69. 33004513 10.1126/science.abb8587

[16] Muzio L , Viotti A, Martino G. Microglia in neuroinflammation and neurodegeneration: from understanding to therapy. Front Neurosci. 2021;15:742065. 34630027 10.3389/fnins.2021.742065 PMC8497816

[17] Ejlerskov P et al Lack of neuronal IFN-β-IFNAR causes Lewy body- and Parkinson’s disease-like dementia. Cell. 2015;163:324–339. 26451483 10.1016/j.cell.2015.08.069 PMC4601085

[18] Escoubas CC et al Type-I-interferon-responsive microglia shape cortical development and behavior. Cell. 2024;187:1936–1954.e24. 38490196 10.1016/j.cell.2024.02.020 PMC11015974

[19] Roy ER et al Type I interferon response drives neuroinflammation and synapse loss in Alzheimer disease. J Clin Invest. 2020;130:1912–1930. 31917687 10.1172/JCI133737 PMC7108898

[20] Roy ER et al Concerted type I interferon signaling in microglia and neural cells promotes memory impairment associated with amyloid β plaques. Immunity. 2022;55:879–894.e6. 35443157 10.1016/j.immuni.2022.03.018 PMC9109419

[21] Hammond TR et al Single-cell RNA sequencing of microglia throughout the mouse lifespan and in the injured brain reveals complex cell-state changes. Immunity. 2019;50:253–271.e6. 30471926 10.1016/j.immuni.2018.11.004 PMC6655561

[22] Gulen MF et al cGAS–STING drives ageing-related inflammation and neurodegeneration. Nature. 2023;620:374–380. 37532932 10.1038/s41586-023-06373-1 PMC10412454

[23] Gunderstofte C et al Nrf2 negatively regulates type I interferon responses and increases susceptibility to herpes genital infection in mice. Front Immunol. 2019;10:2101. 31555293 10.3389/fimmu.2019.02101 PMC6742979

[24] Wyler E et al Single-cell RNA-sequencing of herpes simplex virus 1-infected cells connects NRF2 activation to an antiviral program. Nat Commun. 2019;10:4878. 31653857 10.1038/s41467-019-12894-z PMC6814756

[25] Ryan DG et al Nrf2 activation reprograms macrophage intermediary metabolism and suppresses the type I interferon response. iScience. 2022;25:103827. 35198887 10.1016/j.isci.2022.103827 PMC8844662

[26] Thimmulappa RK et al Nrf2 is a critical regulator of the innate immune response and survival during experimental sepsis. J Clin Invest. 2006;116:984–995. 16585964 10.1172/JCI25790 PMC1421348

[27] Kobayashi EH et al Nrf2 suppresses macrophage inflammatory response by blocking proinflammatory cytokine transcription. Nat Commun. 2016;7:11624. 27211851 10.1038/ncomms11624 PMC4879264

[28] Olagnier D et al Cellular oxidative stress response controls the antiviral and apoptotic programs in dengue virus-infected dendritic cells. PLoS Pathog. 2014;10:e1004566. 25521078 10.1371/journal.ppat.1004566 PMC4270780

[29] Olagnier D et al Activation of Nrf2 signaling augments vesicular stomatitis virus oncolysis via autophagy-driven suppression of antiviral immunity. Mol Ther. 2017;25:1900–1916. 28527723 10.1016/j.ymthe.2017.04.022 PMC5542709

[30] Olagnier D et al SARS-CoV2-mediated suppression of NRF2-signaling reveals potent antiviral and anti-inflammatory activity of 4-octyl-itaconate and dimethyl fumarate. Nat Commun. 2020;11:5419. 33009401 10.1038/s41467-020-18764-3 PMC7532469

[31] Olagnier D et al Nrf2 negatively regulates STING indicating a link between antiviral sensing and metabolic reprogramming. Nat Commun. 2018;9:3506. 30158636 10.1038/s41467-018-05861-7 PMC6115435

[32] Wu M , Fan Y, Li L, Yuan J. Bi-directional regulation of type I interferon signaling by heme oxygenase-1. iScience. 2024;27:109185. 38420586 10.1016/j.isci.2024.109185 PMC10901085

[33] Hubbs AF et al Vacuolar leukoencephalopathy with widespread astrogliosis in mice lacking transcription factor Nrf2. Am J Pathol. 2007;170:2068–2076. 17525273 10.2353/ajpath.2007.060898 PMC1899457

[34] Jazwa A et al Pharmacological targeting of the transcription factor NRf2 at the basal ganglia provides disease modifying therapy for experimental parkinsonism. Antioxid Redox Signal. 2011;14:2347–2360. 21254817 10.1089/ars.2010.3731

[35] Rojo AI et al Nrf2 regulates microglial dynamics and neuroinflammation in experimental Parkinson’s disease. Glia. 2010;58:588–598. 19908287 10.1002/glia.20947

[36] Giesert F et al Expression analysis of Lrrk1, Lrrk2 and Lrrk2 splice variants in mice. PLoS One. 2013;8:e63778. 23675505 10.1371/journal.pone.0063778 PMC3651128

[37] Rocha EM , Keeney MT, Di Maio R, De Miranda BR, Greenamyre JT. LRRK2 and idiopathic Parkinson’s disease. Trends Neurosci. 2022;45:224–236. 34991886 10.1016/j.tins.2021.12.002 PMC8854345

[38] Kluss JH , Mamais A, Cookson MR. LRRK2 links genetic and sporadic Parkinson’s disease. Biochem Soc Trans 2019;47:651–661. 30837320 10.1042/BST20180462 PMC6563926

[39] Smith WW et al Kinase activity of mutant LRRK2 mediates neuronal toxicity. Nat Neurosci. 2006;9:1231–1233. 16980962 10.1038/nn1776

[40] Rui Q et al LRRK2 contributes to secondary brain injury through a p38/drosha signaling pathway after traumatic brain injury in rats. Front Cell Neurosci. 2018;12:51. 29545743 10.3389/fncel.2018.00051 PMC5837969

[41] Hwang JA , Choi SK, Kim SH, Kim DW. Pharmacological inhibition of LRRK2 exhibits neuroprotective activity in mouse photothrombotic stroke model. Exp Neurobiol. 2024;33:36–45. 38471803 10.5607/en23023 PMC10938073

[42] Cao J et al Leucine-rich repeat kinase 2 aggravates secondary brain injury induced by intracerebral hemorrhage in rats by regulating the P38 MAPK/Drosha pathway. Neurobiol Dis. 2018;119:53–64. 30048803 10.1016/j.nbd.2018.07.024

[43] Puccini JM et al Leucine-rich repeat kinase 2 modulates neuroinflammation and neurotoxicity in models of human immunodeficiency virus 1-associated neurocognitive disorders. J Neurosci. 2015;35:5271–5283. 25834052 10.1523/JNEUROSCI.0650-14.2015 PMC4381000

[44] Filippone A et al LRRK2 inhibition by PF06447475 antagonist modulates early neuronal damage after spinal cord trauma. Antioxidants. 2022;11:1634. 36139708 10.3390/antiox11091634 PMC9495377

[45] Weindel CG et al LRRK2 maintains mitochondrial homeostasis and regulates innate immune responses to Mycobacterium tuberculosis. Elife. 2020;9:e51071. 10.7554/eLife.51071 PMC7159881 32057291

[46] Lian H , Roy E, Zheng H. Protocol for primary microglial culture preparation. Bio Protoc. 2016;6:e1989. 10.21769/BioProtoc.1989 PMC5669279 29104890

[47] Van den Bossche J , Baardman J, de Winther MPJ. Metabolic characterization of polarized M1 and M2 bone marrow-derived macrophages using real-time extracellular flux analysis. J Vis Exp. 2015;105:53424. 10.3791/53424 PMC4692751 26649578

[48] Quan Y et al Association between the risk and severity of Parkinson’s disease and plasma homocysteine, vitamin B12 and folate levels: a systematic review and meta-analysis. Front Aging Neurosci. 2023;15:1254824. 37941998 10.3389/fnagi.2023.1254824 PMC10628521

[49] Krawczyk MC , Godoy M, Vander P, Zhang AJ, Zhang Y. Loss of Serpin E2 alters antimicrobial gene expression by microglia but not astrocytes. Neurosci Lett. 2023;811:137354. 37348749 10.1016/j.neulet.2023.137354 PMC11473033

[50] Asheuer M et al Human CD34+ cells differentiate into microglia and express recombinant therapeutic protein. Proc Natl Acad Sci USA. 2004;101:3557–3562. 14990803 10.1073/pnas.0306431101 PMC373501

[51] Caillet-Boudin ML et al Brain pathology in myotonic dystrophy: When tauopathy meets spliceopathy and RNAopathy. Front Mol Neurosci. 2014;6:57. 24409116 10.3389/fnmol.2013.00057 PMC3885824

[52] Tzeng SF , De Vellis J. Id1, Id2, and Id3 gene expression in neural cells during development. Glia. 1998;24:372–381. 9814817 10.1002/(sici)1098-1136(199812)24:4<372::aid-glia2>3.0.co;2-b

[53] Lee AJ , Ashkar AA. The dual nature of type I and type II interferons. Front Immunol. 2018;9:2061. 30254639 10.3389/fimmu.2018.02061 PMC6141705

[54] Ivashkiv LB , Donlin LT. Regulation of type I interferon responses. Nat Rev Immunol. 2014;14:36–49. 24362405 10.1038/nri3581 PMC4084561

[55] Arimoto KI , Miyauchi S, Stoner SA, Fan JB, Zhang DE. Negative regulation of type I IFN signaling. J Leukoc Biol. 2018;103:1099–1116. 10.1002/JLB.2MIR0817-342R 29357192

[56] Tanaka T , Murakami K, Bando Y, Yoshida S. Interferon regulatory factor 7 participates in the M1-like microglial polarization switch. Glia. 2015;63:595–610. 25422089 10.1002/glia.22770

[57] Liddelow SA et al Neurotoxic reactive astrocytes are induced by activated microglia. Nature. 2017;541:481–487. 28099414 10.1038/nature21029 PMC5404890

[58] Wu D et al Type 1 interferons induce changes in core metabolism that are critical for immune function. Immunity. 2016;44:1325–1336. 27332732 10.1016/j.immuni.2016.06.006 PMC5695232

[59] Kelly B , O’Neill LA. Metabolic reprogramming in macrophages and dendritic cells in innate immunity. Cell Res. 2015;25:771–784. 26045163 10.1038/cr.2015.68 PMC4493277

[60] Abdalkader M , Lampinen R, Kanninen KM, Malm TM, Liddell JR. Targeting Nrf2 to suppress ferroptosis and mitochondrial dysfunction in neurodegeneration. Front Neurosci. 2018;12:466. 30042655 10.3389/fnins.2018.00466 PMC6048292

[61] Gumeni S , Papanagnou ED, Manola MS, Trougakos IP. Nrf2 activation induces mitophagy and reverses Parkin/Pink1 knock down-mediated neuronal and muscle degeneration phenotypes. Cell Death Dis. 2021;12:671. 34218254 10.1038/s41419-021-03952-w PMC8254809

[62] Bento-Pereira C , Dinkova-Kostova AT. Activation of transcription factor Nrf2 to counteract mitochondrial dysfunction in Parkinson’s disease. Med Res Rev. 2021;41:785–802. 32681666 10.1002/med.21714

[63] Cvetko F et al Nrf2 is activated by disruption of mitochondrial thiol homeostasis but not by enhanced mitochondrial superoxide production. J Biol Chem. 2021;296:100169. 33298526 10.1074/jbc.RA120.016551 PMC7948991

[64] Baird L , Yamamoto M. The molecular mechanisms regulating the KEAP1-NRF2 pathway. Mol Cell Biol. 2020;40:e00099–20. 32284348 10.1128/MCB.00099-20 PMC7296212

[65] Singh A et al Small molecule inhibitor of NRF2 selectively intervenes therapeutic resistance in KEAP1-deficient NSCLC tumors. ACS Chem Biol. 2016;11:3214–3225. 27552339 10.1021/acschembio.6b00651 PMC5367156

[66] Olayanju A et al Brusatol provokes a rapid and transient inhibition of Nrf2 signaling and sensitizes mammalian cells to chemical toxicity—implications for therapeutic targeting of Nrf2. Free Radic Biol Med. 2015;78:202–212. 25445704 10.1016/j.freeradbiomed.2014.11.003 PMC4291150

[67] Ilieva NM , Hoffman EK, Ghalib MA, Greenamyre JT, De Miranda BR. LRRK2 kinase inhibition protects against Parkinson’s disease-associated environmental toxicants. Neurobiol Dis. 2024;196:106522. 38705492 10.1016/j.nbd.2024.106522 PMC11332574

[68] Lubben N et al LRRK2 kinase inhibition reverses G2019S mutation-dependent effects on tau pathology progression. Transl Neurodegener. 2024;13:13. 38438877 10.1186/s40035-024-00403-2 PMC10910783

[69] West AB. Achieving neuroprotection with LRRK2 kinase inhibitors in Parkinson disease. Exp Neurol. 2017;298:236–245. 28764903 10.1016/j.expneurol.2017.07.019 PMC5693612

[70] Wojewska DN , Kortholt A. Lrrk2 targeting strategies as potential treatment of Parkinson’s disease. Biomolecules. 2021;11:10.3390/biom11081101 PMC8392603 34439767

[71] Taymans JM et al Perspective on the current state of the LRRK2 field. NPJ Parkinsons Dis. 2023;9:104. 37393318 10.1038/s41531-023-00544-7 PMC10314919

[72] Kelly K et al The G2019S mutation in LRRK2 imparts resiliency to kinase inhibition. Exp Neurol. 2018;309:1–13. 30048714 10.1016/j.expneurol.2018.07.012 PMC7041630

[73] Ozelius LJ et al LRRK2 G2019S as a cause of Parkinson’s disease in Ashkenazi Jews. New Engl J Med. 2006;354:424–425. 16436782 10.1056/NEJMc055509

[74] Weindel CG et al Mitochondrial dysfunction promotes alternative gasdermin D-mediated inflammatory cell death and sus-ceptibility to infection.

[75] West AP et al Mitochondrial DNA stress primes the antiviral innate immune response. 10.1038/nature14156 PMC4409480 25642965

[76] Yin W et al; UNC/UMN Baby Connectome Project Consortium. Charting brain functional development from birth to 6 years of age. Nat Hum Behav. 2025;9:1246–1259. 40234630 10.1038/s41562-025-02160-2 PMC12185323

[77] Ben-Yehuda H et al Maternal Type-I interferon signaling adversely affects the microglia and the behavior of the offspring accompanied by increased sensitivity to stress. Mol Psychiatry. 2020;25:1050–1067. 31772304 10.1038/s41380-019-0604-0 PMC7192855

[78] Shah ZA et al Role of reactive oxygen species in modulation of Nrf2 following ischemic reperfusion injury. Neuroscience. 2007;147:53–59. 17507167 10.1016/j.neuroscience.2007.02.066 PMC1961622

[79] Shih AY , Li P, Murphy TH. A small-molecule-inducible Nrf2-mediated antioxidant response provides effective prophylaxis against cerebral ischemia in vivo. J Neurosci. 2005;25:10321–10335. 16267240 10.1523/JNEUROSCI.4014-05.2005 PMC6725780

[80] Chen PC et al Nrf2-mediated neuroprotection in the MPTP mouse model of Parkinson’s disease: Critical role for the astrocyte. Proc Natl Acad Sci U S A. 2009;106:2933–2938. 19196989 10.1073/pnas.0813361106 PMC2650368

[81] Branca C et al Genetic reduction of Nrf2 exacerbates cognitive deficits in a mouse model of Alzheimer’s disease. Hum Mol Genet. 2017;26:4823–4835. 29036636 10.1093/hmg/ddx361 PMC5886066

[82] Todorovic M et al Comprehensive assessment of genetic sequence variants in the antioxidant ‘master regulator’ Nrf2 in idiopathic Parkinson’s disease. PLoS One. 2015;10:e0128030. 10.1371/journal.pone.0128030 PMC4444110 26010367

[83] Gui Y et al NFE2L2 variations reduce antioxidant response in patients with Parkinson disease. Oncotarget. 2016;7:10756–10764. 10.18632/oncotarget.7353 PMC4905436 26887053

[84] Peng S , Chen Y, Wang R, Zhang J. Z-ligustilide provides a neuroprotective effect by regulating the phenotypic polarization of microglia via activating Nrf2-TrxR axis in the Parkinson’s disease mouse model. Redox Biol. 2024;76:103324. 39180982 10.1016/j.redox.2024.103324 PMC11388202

[85] Zhang N et al Nrf2 signaling contributes to the neuroprotective effects of urate against 6-OHDA toxicity. PLoS One. 2014;9:e100286. 24959672 10.1371/journal.pone.0100286 PMC4069024

[86] Nakano-Kobayashi A et al Therapeutics potentiating microglial p21-Nrf2 axis can rescue neurodegeneration caused by neuroinflammation. Sci Adv. 2020;6:eabc1428. 10.1126/sciadv.abc1428 PMC7673758 33188020

[87] Sun W , Shen Y, Xiao H, Li H. Resveratrol attenuates rotenone-induced inflammation and oxidative stress via STAT1 and Nrf2/Keap1/SLC7A11 pathway in a microglia cell line. Pathol Res Pract. 2021;225:153576. 34391968 10.1016/j.prp.2021.153576

[88] Fan W et al NRF2 activation ameliorates blood–brain barrier injury after cerebral ischemic stroke by regulating ferroptosis and inflammation. Sci Rep. 2024;14:5300. 38438409 10.1038/s41598-024-53836-0 PMC10912757

[89] Cui P , Song B, Xia Z, Xu Y. Type I interferon signalling and ischemic stroke: mechanisms and therapeutic potentials. Transl Stroke Res. 2025;16:962–974. 38466560 10.1007/s12975-024-01236-x

[90] Naidu S , Vijayan V, Santoso S, Kietzmann T, Immenschuh S. Inhibition and genetic deficiency of p38 MAPK up-regulates heme oxygenase-1 gene expression via Nrf2. J Immunol. 2009;182:7048–7057. 19454702 10.4049/jimmunol.0900006

[91] Hourihan JM , Moronetti Mazzeo LE, Fernández-Cárdenas LP, Blackwell TK. Cysteine sulfenylation directs IRE-1 to activate the SKN-1/Nrf2 antioxidant response. Mol Cell. 2016;63:553–566. 27540856 10.1016/j.molcel.2016.07.019 PMC4996358

