# Supplementary material for: LRRK2 kinase activity restricts NRF2-dependent mitochondrial protection in microglia
Source: J Immunol. 2025 Sep 4;215(1):vkaf215. doi: 10.1093/jimmun/vkaf215 (PMC12412900; doi:10.1093/jimmun/vkaf215)
Supplement: vkaf215_Supplementary_Data [file vkaf215_supplementary_data.pdf]

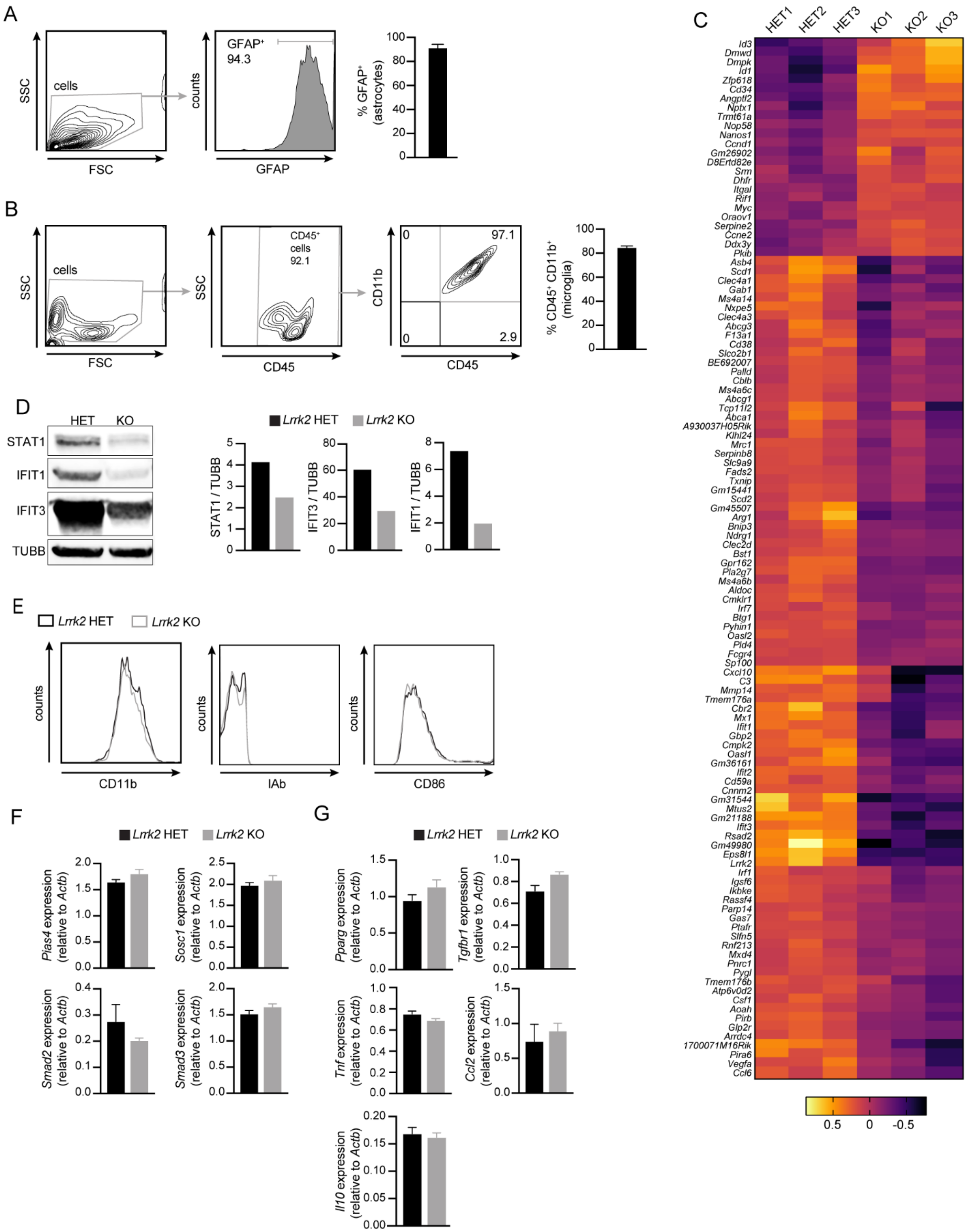
A
SSC
FSC
cells
counts
GFAP
GFAP+ 94.3
% GFAP+ (astrocytes)
B
CD45+ cells 92.1
CD11b
CD45
97.1
2.9
% CD45+ CD11b+ (microglia)
C
HET1 HET2 HET3 KO1 KO2 KO3
Id3
Dmwd
Dmpk
Id1
Zfp618
Cd34
Angptl2
Nptx1
Trmt61a
Nop58
Nanos1
Ccnd1
Gm26902
D8Ertd82e
Srm
Dhfr
Itgal
Rif1
Myc
Oraov1
Serpine2
Ccne2
Ddx3y
Pkib
Asb4
Scd1
Clec4a1
Gab1
Ms4a14
Nxpe5
Clec4a3
Abcg3
F13a1
Cd38
Slco2b1
BE692007
Palld
Cblb
Ms4a6c
Abcg1
Tcp11l2
Abca1
A930037H05Rik
Klhl24
Mrc1
Serpinb8
Slc9a9
Fads2
Txnip
Gm15441
Scd2
Gm45507
Arg1
Bnip3
Ndrg1
Clec2d
Bst1
Gpr162
Pla2g7
Ms4a6b
Aldoc
Cmklr1
Irf7
Btg1
Pyhin1
Oasl2
Pld4
Fcgr4
Sp100
Cxcl10
C3
Mmp14
Tmem176a
Cbr2
Mx1
Ifit1
Gbp2
Cmpk2
Oasl1
Gm36161
Ifit2
Cd59a
Cnnm2
Gm31544
Mtus2
Gm21188
Ifit3
Rsad2
Gm49980
Eps8l1
Lrrk2
Irf1
Igsf6
Ikbke
Rassf4
Parp14
Gas7
Ptafr
Slfn5
Rnf213
Mxd4
Pnrc1
Pygl
Tmem176b
Atp6v0d2
Csf1
Aoah
Pirb
Glp2r
Arrdc4
1700071M16Rik
Pira6
Vegfa
Ccl6
0.5 0 -0.5
D
HET KO
STAT1
IFIT1
IFIT3
TUBB
Lrrk2 HET Lrrk2 KO
STAT1 / TUBB
IFIT3 / TUBB
IFIT1 / TUBB
E
Lrrk2 HET Lrrk2 KO
counts
CD11b
IAb
CD86
F
Lrrk2 HET Lrrk2 KO
Pias4 expression (relative to Actb)
Sosc1 expression (relative to Actb)
Smad2 expression (relative to Actb)
Smad3 expression (relative to Actb)
G
Lrrk2 HET Lrrk2 KO
Pparg expression (relative to Actb)
Tgfbr1 expression (relative to Actb)
Tnf expression (relative to Actb)
Ccl2 expression (relative to Actb)
Il10 expression (relative to Actb)

Figure S1

Supplemental Figures

Figure S1: Gating strategy and transcript profile of microglial cells (**A.**) GFAP expression and cell percentages of astrocyte populations analyzed by flow cytometry. (**B.**) CD45 and CD11b expression and cell percentages of microglial populations analyzed by flow cytometry. (**C.**) Heatmap of significant differentially expressed genes between *Lrrk2* KO microglia and HET controls. (**D.**) Protein levels of ISGs, STAT1, IFIT1, and IFIT3, compared to TUBB in *Lrrk2* KO and HET microglia measured by western blot. (**E.**) CD11b, IAb, CD86 expression of *Lrrk2* KO and HET microglia measured by flow cytometry. (**F.**) Transcript levels of negative regulators of the type I IFN response *Pias4*, *Sosc1*, *Smad2*, *Smad3*, in *Lrrk2* KO and HET microglia measured by qRT-PCR. (**G.**) The same as in (D), but M1 vs M2 macrophage markers *Tnf*, *Ccl2*, *Pparg*, *Tgfbr1*, and *Il10*. Two-tailed Student's t-test was used to determine statistical significance. *p<0.05, **p<0.01, ***p<0.005.

A

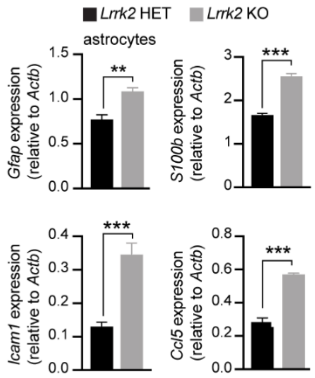

Lrrk2 HET
Lrrk2 KO
astrocytes
Gfap expression (relative to Actb)
S100b expression (relative to Actb)
Icam1 expression (relative to Actb)
Ccl5 expression (relative to Actb)
**
***
***
***

Figure S2

Figure S2: Loss of LRRK2 in astrocytes upregulates activation-associated factors

Transcript levels of astrocyte activation and ISG associated genes *Gfap*, *S100b*, *Icam1*, *Ccl5*, in *Lrrk2* KO and HET astrocytes measured by qRT-PCR. Two-tailed Student's t-test was used to determine statistical significance. *p<0.05, **p<0.01, ***p<0.005.

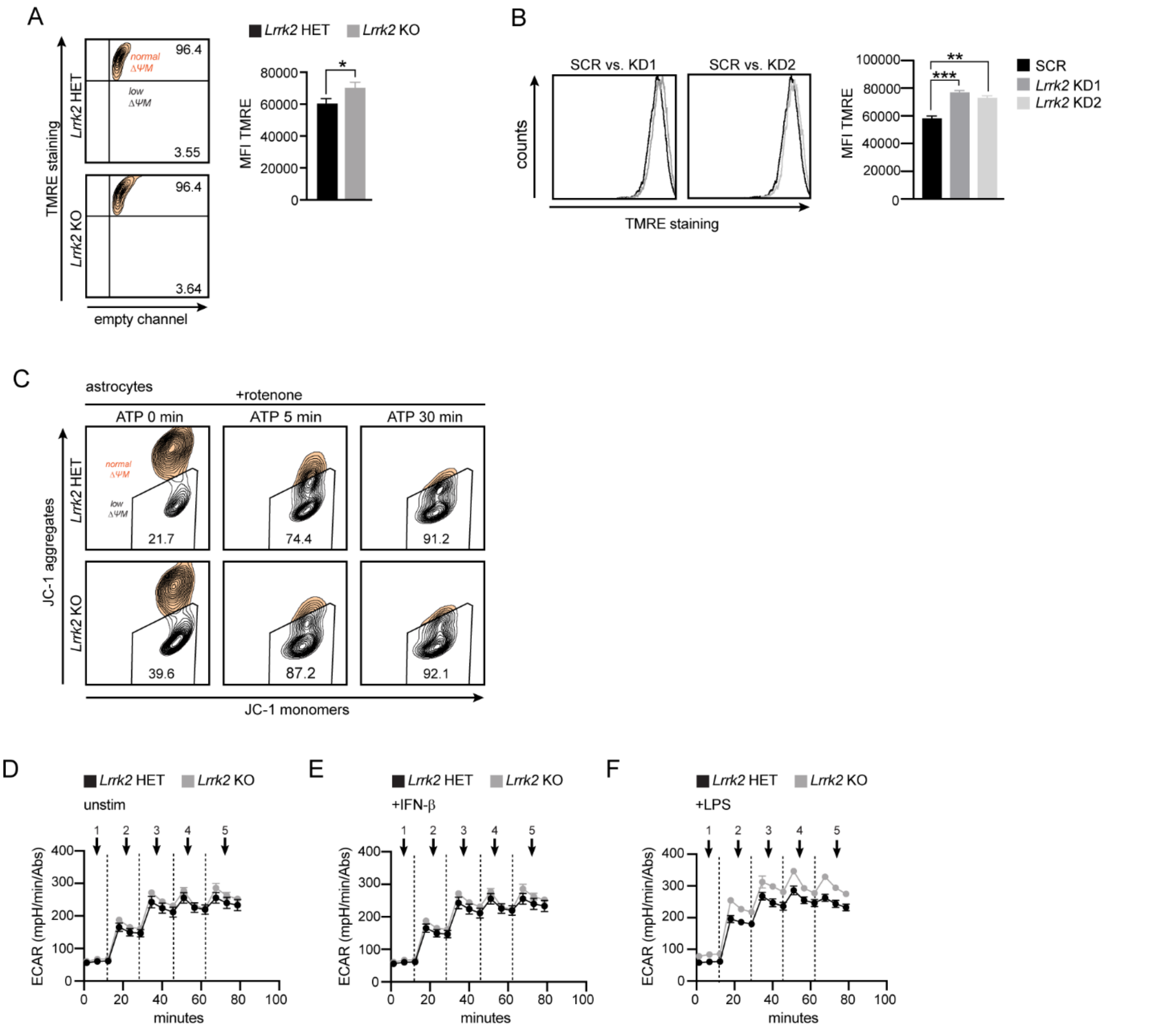
A
Lrrk2 HET
Lrrk2 KO
TMRE staining
empty channel
normal ΔΨM
low ΔΨM
96.4
3.55
96.4
3.64
MFI TMRE
B
SCR vs. KD1
SCR vs. KD2
counts
TMRE staining
SCR
Lrrk2 KD1
Lrrk2 KD2
C
astrocytes
+rotenone
ATP 0 min
ATP 5 min
ATP 30 min
JC-1 aggregates
JC-1 monomers
21.7
74.4
91.2
39.6
87.2
92.1
D
unstim
E
+IFN-β
F
+LPS
ECAR (mpH/min/Abs)
minutes

Figure S3

Figure S3: Microglial glycolytic flux is not impacted by a loss of LRRK2
(**A.**) TMRE staining of mitochondrial membrane potential in *Lrrk2* KO and HET microglia measured by flow cytometry. (**B.**) The same as in (A) but cells were SIMA9 microglia *Lrrk2* KD vs. SCR. (**D.**) Extracellular acidification rate (ECAR), a measurement for glycolysis, of unstimulated *Lrrk2* KO and HET microglia (**C.**) JC-1 staining of mitochondrial membrane potential in *Lrrk2* KO and HET astrocytes measured by flow cytometry. Cells were treated with 2.5 µM rotenone for 3 hrs. followed by 5 µM ATP for 5 and 30 min. (**D.**) Extracellular acidification rate (ECAR), a proxy of glycolysis, in resting *Lrrk2* KO and HET microglia measured by the seahorse bioanalyzer mito-stress test. (**E.**) The same as in (D), but cells were treated for 16 hrs. with 100 IU IFNβ (**F.**) The same as in (D) and (E), but cells were treated for 16 hrs. with 10 ng/mL LPS. Two-tailed Student's t-test or One way ANOVA with Sidak's multiple comparisons was used to determine statistical significance. *p<0.05, **p<0.01, ***p<0.005.

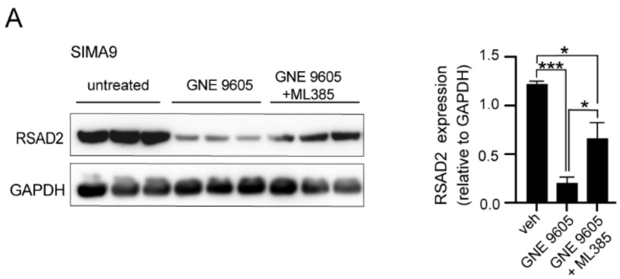
A
SIMA9
untreated
GNE 9605
GNE 9605
+ML385
RSAD2
GAPDH
RSAD2 expression
(relative to GAPDH)
1.5
1.0
0.5
0.0
***
*
*
veh
GNE 9605
GNE 9605
+ ML385

Figure S4

Figure S4: ISG reduction caused by LRRK2 kinase inhibition is dependent on NRF2. Protein levels as measured by western blot for RSAD2 in SIMA9 cells treated for 72 hrs with either vehicle control (untreated), 10μM GNE9605, or 10 μM GNE9605 and 5 μM ML385. RSAD2 expression was normalized to GAPDH, quantification on right. One way ANOVA with Sidak's multiple comparisons was used to determine statistical significance. *$p<0.05$, **$p<0.01$, ***$p<0.005$.

Table S1 Genes differentially expressed in *Lrrk2* KO microglial cells

Symbol	ensembl_gene_id	baseMean	log2FoldChange	pvalue	padj
Mx1	ENSMUSG00000000386	355.0362219	-0.42058263	3.58655137863256e-10	1.17E-06
Ccl6	ENSMUSG00000018927	22553.17965	-0.350855155	2.5185923695444e-11	1.23E-07
Ifit3	ENSMUSG00000074896	285.532859	-0.395319665	5.65742322674163e-09	1.38E-05
Gm21188	ENSMUSG00000095609	211.8625126	-0.421695307	6.08748286144667e-10	1.78E-06
Rsad2	ENSMUSG00000020641	806.0608255	-0.658170181	6.26996678774652e-26	1.83E-21
Cmklr1	ENSMUSG00000042190	948.8821906	-0.326403449	1.16833888215168e-08	2.63E-05
Pld4	ENSMUSG00000052160	5294.609927	-0.242086376	1.9977631869087e-08	4.18E-05
Ccnd1	ENSMUSG00000070348	17010.94939	0.250067578	2.55385991983937e-08	4.67E-05
Gm45507	ENSMUSG00000109881	504.3784983	-0.369064373	2.45660662818828e-08	4.67E-05
Slfn5	ENSMUSG00000054404	3080.019112	-0.252509212	2.76260152655099e-08	4.76E-05
Cmpk2	ENSMUSG00000020638	458.6344933	-0.443936046	6.80182407384014e-12	4.98E-08
Ifit2	ENSMUSG00000045932	1000.898804	-0.405657463	5.35661668421908e-13	5.22E-09
Dmpk	ENSMUSG00000030409	263.9557634	0.375287899	3.72049923407784e-08	5.73E-05
Btg1	ENSMUSG00000036478	2940.73431	-0.255217741	3.54360185847417e-08	5.73E-05
Oasl2	ENSMUSG00000029561	1300.892578	-0.29090249	4.03667444884198e-08	5.91E-05
Id1	ENSMUSG00000042745	479.5233192	0.520492105	4.33095996897773e-15	6.34E-11
Nanos1	ENSMUSG00000072437	892.1612332	0.318923837	4.73588231335272e-08	6.60E-05
Pirb	ENSMUSG00000058818	1438.712316	-0.356412528	1.71198151624915e-10	7.16E-07
Pla2g7	ENSMUSG00000023913	629.623263	-0.363393604	2.92832148118868e-09	7.79E-06
Lrrk2	ENSMUSG00000036273	244.1741241	-0.431469609	2.48837680521509e-10	9.10E-07
Angptl2	ENSMUSG00000004105	581.0879941	0.421664463	1.61118439857454e-11	9.43E-08
Clec4a1	ENSMUSG00000049037	456.7868279	-0.353512417	8.93814275263235e-08	0.000118881
Ms4a6b	ENSMUSG00000024677	1337.879291	-0.296880386	1.3593193882623e-07	0.000170324
Scd1	ENSMUSG00000037071	552.5644554	-0.357092531	1.45521182079285e-07	0.000170324
Txnip	ENSMUSG00000038393	8894.071897	-0.239906272	1.42114754678581e-07	0.000170324
Ptafr	ENSMUSG00000056529	2479.001778	-0.244832145	1.52419862340808e-07	0.000171537
Ikbke	ENSMUSG00000042349	3541.212882	-0.270976815	2.92169959713816e-07	0.000316636
Bnip3	ENSMUSG00000078566	1037.698041	-0.310924237	4.40900429960166e-07	0.000460757
Dmwd	ENSMUSG00000030410	254.2082643	0.343263986	4.7920987346982e-07	0.000483523
Scd2	ENSMUSG00000025203	20420.28949	-0.24945602	5.93654601213745e-07	0.00057318
Mrc1	ENSMUSG00000026712	30693.89323	-0.25426338	6.07244277879077e-07	0.00057318
Parp14	ENSMUSG00000034422	3797.070872	-0.213116411	8.37519920076134e-07	0.000765833
Nop58	ENSMUSG00000026020	1802.706518	0.245128191	9.95881054164405e-07	0.000871805
Gm15441	ENSMUSG00000074398	2518.199549	-0.269143454	1.01299944892024e-06	0.000871805
Gas7	ENSMUSG00000033066	3661.040014	-0.238471488	1.24083673402614e-06	0.001037375
Aoah	ENSMUSG00000021322	1832.147085	-0.257745302	2.12226276088558e-06	0.001724987
Id3	ENSMUSG00000007872	256.6974305	0.320073293	2.34647593258769e-06	0.001855682
Zfp618	ENSMUSG00000028358	234.823649	0.318276015	2.97716213362874e-06	0.002280966
Rnf213	ENSMUSG00000070327	8520.567628	-0.23157677	3.04014518805654e-06	0.002280966
F13a1	ENSMUSG00000039109	1767.851658	-0.28352872	3.30014107001378e-06	0.002414136
Atp6v0d2	ENSMUSG00000028238	2266.231382	-0.259509923	3.71354853075409e-06	0.002650296

Abcg1	ENSMUSG00000024030	2850.754606	-0.223302992	4.01444841799725e-06	0.002669699
Rassf4	ENSMUSG00000042129	8398.128165	-0.242411574	3.93479723714927e-06	0.002669699
Ms4a6c	ENSMUSG00000079419	1969.443744	-0.236117792	3.9006976145246e-06	0.002669699
Fads2	ENSMUSG00000024665	2138.480125	-0.240782864	4.87794637898809e-06	0.003171858
BE692007	ENSMUSG00000099757	1225.239001	-0.26040228	5.87503097059377e-06	0.003737158
Gm49980	ENSMUSG00000117465	56.3049163	-0.245902029	8.59069689405246e-06	0.005348349
Serpine2	ENSMUSG00000026249	725.1237858	0.267925232	9.07327764739681e-06	0.005531108
LTO1	ENSMUSG00000031072	6321.026173	0.211615588	1.22783421382309e-05	0.007332175
Asb4	ENSMUSG00000042607	217.8974143	-0.296700083	1.31295060544606e-05	0.00768365
Anxa6	ENSMUSG00000018340	3840.528898	-0.185507582	1.39021349151755e-05	0.007976282
Ccne2	ENSMUSG00000028212	948.5579389	0.251344269	1.57432022401697e-05	0.008858882
C3	ENSMUSG00000024164	215.8037746	-0.288369384	1.69461717699334e-05	0.009355886
Cfp	ENSMUSG00000001128	7104.050274	-0.191775675	1.73793876075679e-05	0.009417375
Vegfa	ENSMUSG00000023951	786.6521523	-0.283625228	1.8791418354417e-05	0.009818852
Sp100	ENSMUSG00000026222	2036.133682	-0.203732084	1.87141504981468e-05	0.009818852
Serpinb8	ENSMUSG00000026315	1111.941111	-0.237794411	2.19309121430956e-05	0.011258253
Ms4a14	ENSMUSG00000099398	519.5861955	-0.276842577	2.39308628731499e-05	0.01207312
Dhfr	ENSMUSG00000021707	1030.542086	0.243019183	2.65051006733531e-05	0.013145182
Slc9a9	ENSMUSG00000031129	2429.584451	-0.222602804	2.72096449191921e-05	0.01326969
Fcgr4	ENSMUSG00000059089	1455.216766	-0.214242616	2.83027100526752e-05	0.013576485
Igsf6	ENSMUSG00000035004	2473.395497	-0.242845628	3.26545351501954e-05	0.015411361
Pygl	ENSMUSG00000021069	1471.440652	-0.218216299	4.03522832131691e-05	0.018742034
Itgal	ENSMUSG00000030830	1312.77329	0.221063589	4.17853789334909e-05	0.019104406
Cbr2	ENSMUSG00000025150	115.3288737	-0.26363653	4.43145713052589e-05	0.019949056
Mxd4	ENSMUSG00000037235	1506.471027	-0.218210136	5.37810165710894e-05	0.023843732
Ifit1	ENSMUSG00000034459	220.0832689	-0.274553134	5.61465288197333e-05	0.024520949
Cxcl10	ENSMUSG00000034855	177.0435405	-0.261242976	5.76118770533664e-05	0.024790899
Slfn1	ENSMUSG00000078763	18.4658387	-0.15040924	6.22234971195828e-05	0.026387272
Gbp2	ENSMUSG00000028270	247.8424431	-0.272118926	6.57785897124777e-05	0.027399352
Cd38	ENSMUSG00000029084	1647.623053	-0.247250417	6.64828273647452e-05	0.027399352
Cd83	ENSMUSG00000015396	3619.689696	-0.175908053	6.92662592428115e-05	0.02815
Fabp5	ENSMUSG00000027533	14739.27207	-0.153203728	7.41588079441429e-05	0.029725491
Smc2	ENSMUSG00000028312	4259.910888	0.162640104	7.79333521367366e-05	0.030816322
Ifi204	ENSMUSG00000073489	2589.253507	-0.182005873	8.08883687998581e-05	0.031558327
Hexb	ENSMUSG00000021665	17923.8208	0.155695187	8.25889722921444e-05	0.031797841
Ifit1bl1	ENSMUSG00000079339	43.64842645	-0.192413105	8.54438873658784e-05	0.032469787
Pltp	ENSMUSG00000017754	1840.493309	-0.197314357	9.30145122548143e-05	0.034467567
Ahnak	ENSMUSG00000069833	17354.28388	-0.180249831	9.30568948705226e-05	0.034467567
Cd34	ENSMUSG00000016494	184.4509589	0.265265689	9.49729322427011e-05	0.034686046
Igf1	ENSMUSG00000020053	28320.47759	0.175378879	9.80752745370812e-05	0.034686046
P2rx4	ENSMUSG00000029470	3419.595219	-0.183387672	9.83883607357534e-05	0.034686046
Trmt61a	ENSMUSG00000060950	262.3127	0.2652115	9.62397524733134e-05	0.034686046
Gm31544	ENSMUSG00000114375	58.31191434	-0.218708175	0.000100124	0.034877564
Serinc3	ENSMUSG00000017707	26398.48018	-0.139883621	0.000111811	0.038490485
Rif1	ENSMUSG00000036202	1452.788769	0.211252169	0.000118555	0.040337658

Tfrc	ENSMUSG00000022797	2813.873514	0.196295767	0.000120966	0.04065477
Gpr162	ENSMUSG00000038390	234.7102116	-0.261758339	0.000122266	0.04065477
Abca1	ENSMUSG00000015243	6012.702116	-0.259436686	0.000126627	0.041551425
Myc	ENSMUSG00000022346	1129.127198	0.215511945	0.000130551	0.041551425
Tmem176a	ENSMUSG00000023367	375.2132555	-0.260250473	0.000130703	0.041551425
Rap2a	ENSMUSG00000051615	4853.806097	0.165953277	0.000132063	0.041551425
Ddx3y	ENSMUSG00000069045	1174.830927	0.227482082	0.000129863	0.041551425
Azin1	ENSMUSG00000037458	5846.109872	0.184156137	0.000150815	0.046946892
Cnnm2	ENSMUSG00000064105	298.0105986	-0.254609312	0.000154906	0.04771263
Unc93b1	ENSMUSG00000036908	8900.601603	-0.151322068	0.000158525	0.048318807

*Major significantly upregulated or downregulated genes in *Lrrk2* KO microglia compared to *Lrrk2* HET controls. Significant genes are based on adjusted p value. Table is arranged by adjusted p value low>high values.

Table S2 Genes differentially expressed in *Lrrk2* KO macrophages

Symbol	ensemble_gene_id	AveExpr	log2FoldChange	pvalue	padj
Apoe	ENSMUSG00000002985.16	8.602786101	-0.887548239	9.42E-29	1.22E-24
Gpnmb	ENSMUSG00000029816.10	12.88448326	-0.183417163	1.15E-24	7.41E-21
Lyz2	ENSMUSG00000069516.7	12.41967256	-0.2386303	9.72E-24	4.19E-20
Ctsd	ENSMUSG00000007891.16	11.50040162	-0.22825035	6.31E-20	2.04E-16
B2m	ENSMUSG00000060802.8	10.32029946	0.327332135	8.49E-20	2.19E-16
Spp1	ENSMUSG00000029304.14	11.08469533	-0.218255141	2.14E-19	4.60E-16
Ly6e	ENSMUSG00000022587.14	9.202184539	0.605711513	4.40E-19	8.13E-16
Oas3	ENSMUSG00000032661.9	8.244564645	0.862492625	6.70E-19	1.08E-15
Irf7	ENSMUSG00000025498.15	7.021074849	1.644682004	8.53E-19	1.22E-15
Sdc3	ENSMUSG00000025743.14	9.633388027	0.343598384	1.53E-18	1.98E-15
Oasl2	ENSMUSG00000029561.17	7.829559357	1.294251134	2.46E-17	2.90E-14
Tgfbi	ENSMUSG00000035493.9	9.97121666	0.292512058	2.78E-17	3.00E-14
Ly6a	ENSMUSG00000075602.10	6.501599979	0.917407668	6.15E-17	6.12E-14
Rtp4	ENSMUSG00000033355.6	6.925363128	0.962098875	1.48E-16	1.37E-13
Sp100	ENSMUSG00000026222.16	7.412486328	0.85347048	2.91E-16	2.51E-13
Ctsb	ENSMUSG00000021939.7	13.06265012	-0.131662771	3.67E-16	2.97E-13
Ifitm3	ENSMUSG00000025492.6	8.521326908	0.507346385	5.97E-16	4.54E-13
H2-K1	ENSMUSG00000061232.15	9.581370132	0.327481821	1.54E-15	1.10E-12
Psap	ENSMUSG00000004207.14	12.37502908	-0.090859773	9.69E-15	6.59E-12
Ifi27l2a	ENSMUSG00000079017.3	6.158303937	0.902675155	1.06E-14	6.83E-12
Rnf213	ENSMUSG00000070327.14	10.43845154	1.036800188	1.30E-14	8.01E-12
Dhx58	ENSMUSG00000017830.15	6.486693161	1.111126794	1.55E-14	9.13E-12
Oas2	ENSMUSG00000032690.16	6.612651039	1.005834224	2.44E-14	1.37E-11
Lgals3bp	ENSMUSG00000033880.11	9.064799235	0.39905743	2.57E-14	1.38E-11
Ifi204	ENSMUSG00000073489.5	7.885419181	1.020594144	2.94E-14	1.52E-11
Ube2l6	ENSMUSG00000027078.14	6.465780919	0.876077112	6.76E-14	3.36E-11
Helz2	ENSMUSG00000027580.17	8.181885461	0.824148145	1.98E-13	9.50E-11
Mfge8	ENSMUSG00000030605.15	7.153092501	-0.496234867	3.17E-13	1.46E-10
Isg15	ENSMUSG00000035692.6	6.404358044	1.567440143	4.99E-13	2.22E-10
Tap1	ENSMUSG00000037321.17	7.186013887	0.776099574	6.49E-13	2.79E-10
Trim30a	ENSMUSG00000030921.17	7.864275951	0.851550047	9.22E-13	3.84E-10
H2-D1	ENSMUSG00000073411.11	10.50180246	0.185459049	1.26E-12	5.10E-10
Zbp1	ENSMUSG00000027514.14	5.03358828	2.038106709	1.46E-12	5.72E-10
Mndal	ENSMUSG00000090272.7	7.288882374	0.989632934	2.54E-12	9.60E-10
Fcgr1	ENSMUSG00000015947.10	7.045884238	0.755800286	2.60E-12	9.60E-10
Stat2	ENSMUSG00000040033.15	7.913240018	0.783903662	2.76E-12	9.91E-10
Igf1	ENSMUSG00000020053.18	8.765725268	-0.326380132	2.85E-12	9.97E-10
Samd9l	ENSMUSG00000047735.14	8.328416055	0.622551816	4.86E-12	1.65E-09

Ddx58	ENSMUSG00000040296.15	7.712707635	0.948495504	6.26E-12	2.08E-09
Parp12	ENSMUSG00000038507.6	7.327221552	0.755902769	1.36E-11	4.38E-09
Slfn2	ENSMUSG00000072620.3	8.626285325	0.497021535	2.05E-11	6.31E-09
Lgals9	ENSMUSG00000001123.15	7.705375985	0.451073067	2.01E-11	6.31E-09
Bst2	ENSMUSG00000046718.7	7.357086604	0.494175767	2.17E-11	6.51E-09
H2-Q4	ENSMUSG00000035929.11	6.419240067	0.651045708	4.69E-11	1.38E-08
Cat	ENSMUSG00000027187.10	8.654717516	-0.258269329	7.64E-11	2.19E-08
H2-T24	ENSMUSG00000053835.17	5.125758469	1.092848529	9.64E-11	2.68E-08
Epsti1	ENSMUSG00000022014.14	5.849337623	0.844388555	9.76E-11	2.68E-08
Xaf1	ENSMUSG00000040483.15	5.678993026	1.019314035	1.38E-10	3.64E-08
Dtx3l	ENSMUSG00000049502.16	7.762623888	0.719691302	1.38E-10	3.64E-08
Treml2	ENSMUSG00000071068.6	4.087555317	1.20049889	1.42E-10	3.68E-08
Selenop	ENSMUSG00000064373.11	8.493502295	-0.28787493	1.49E-10	3.77E-08
Gm42031	ENSMUSG00000110386.1	4.3449232	-1.133522654	1.54E-10	3.83E-08
Adar	ENSMUSG00000027951.16	7.724805718	0.529036478	1.58E-10	3.85E-08
Ifi211	ENSMUSG00000026536.9	6.006454576	1.511280407	1.87E-10	4.48E-08
Slfn5	ENSMUSG00000054404.12	8.051679178	1.026597346	2.63E-10	6.17E-08
Oas1a	ENSMUSG00000052776.10	6.878440031	0.597358006	2.99E-10	6.89E-08
Stat1	ENSMUSG00000026104.14	6.447058141	0.712604768	3.12E-10	7.08E-08
Ifi203	ENSMUSG00000039997.16	7.962560751	0.839060166	3.21E-10	7.13E-08
Eif2ak2	ENSMUSG00000024079.4	7.742045238	0.54812196	3.26E-10	7.13E-08
Parp14	ENSMUSG00000034422.14	9.464675665	0.827717801	4.62E-10	9.95E-08
Fabp5	ENSMUSG00000027533.10	7.159937758	-0.411099941	4.96E-10	1.05E-07
Gm1966	ENSMUSG00000073902.5	4.697355035	1.505523665	5.36E-10	1.12E-07
Hvcn1	ENSMUSG00000064267.13	6.706182104	-0.46403692	5.88E-10	1.21E-07
Nlrc5	ENSMUSG00000074151.12	6.281049371	0.625529654	8.42E-10	1.70E-07
Sp110	ENSMUSG00000070034.13	6.32453391	0.702205375	9.27E-10	1.84E-07
Ifi35	ENSMUSG00000010358.13	5.919162734	0.758599761	1.08E-09	2.11E-07
Irgm1	ENSMUSG00000046879.6	7.847586726	0.903884951	1.12E-09	2.16E-07
Samhd1	ENSMUSG00000027639.16	8.582959008	0.456876813	1.21E-09	2.26E-07
Stab1	ENSMUSG00000042286.13	9.528048466	-0.190413999	1.21E-09	2.26E-07
Psmb8	ENSMUSG00000024338.14	7.359412128	0.436612762	1.28E-09	2.37E-07
Usp18	ENSMUSG00000030107.10	6.616363834	1.418716724	1.49E-09	2.72E-07
Mpeg1	ENSMUSG00000046805.9	11.7076468	-0.103507934	1.81E-09	3.24E-07
Pld3	ENSMUSG00000003363.15	8.303279186	-0.259804359	1.98E-09	3.50E-07
Ddx60	ENSMUSG00000037921.15	4.973826697	1.528841155	2.69E-09	4.69E-07
Trafd1	ENSMUSG00000042726.14	7.493367936	0.576850573	2.72E-09	4.69E-07
Ogfr	ENSMUSG00000049401.10	7.006318168	0.52628446	2.95E-09	5.01E-07
Znfx1	ENSMUSG00000039501.14	8.968408889	0.532374679	2.99E-09	5.02E-07
Csf1r	ENSMUSG00000024621.15	11.04089392	0.088474624	4.28E-09	7.09E-07
Siglec1	ENSMUSG00000027322.11	7.66329667	0.373747558	4.90E-09	8.01E-07
Cd74	ENSMUSG00000024610.14	6.670478944	0.463496668	5.12E-09	8.27E-07
Ctnnd2	ENSMUSG00000022240.9	4.10437835	1.049954419	5.52E-09	8.81E-07
Ifit3	ENSMUSG00000074896.3	7.453705569	1.626101468	7.59E-09	1.20E-06
Tor3a	ENSMUSG00000060519.11	7.634425915	0.503470398	8.83E-09	1.37E-06

Pltp	ENSMUSG00000017754.13	6.99217619	0.401521059	9.69E-09	1.49E-06
Ifi47	ENSMUSG00000078920.2	5.898369283	1.32265413	9.95E-09	1.51E-06
Rap2a	ENSMUSG00000051615.13	7.591528448	-0.264797107	1.01E-08	1.51E-06
Parp9	ENSMUSG00000022906.15	7.334287498	0.565671626	1.17E-08	1.74E-06
Slfn8	ENSMUSG00000035208.15	7.354517536	0.905293498	1.36E-08	2.00E-06
Trim30d	ENSMUSG00000057596.13	5.666579361	0.934432683	1.52E-08	2.21E-06
Ifi207	ENSMUSG00000073490.10	8.606256268	0.570471165	1.58E-08	2.27E-06
Rn18s-rs5	ENSMUSG00000106106.2	5.41354175	0.957908881	1.93E-08	2.74E-06
Irf9	ENSMUSG00000002325.14	6.387327622	0.464528449	2.30E-08	3.23E-06
Ms4a6b	ENSMUSG00000024677.13	5.458805524	0.789662718	2.37E-08	3.26E-06
Itga6	ENSMUSG00000027111.15	8.642319838	0.201004493	2.36E-08	3.26E-06
Itgb3	ENSMUSG00000020689.4	4.880576667	-0.601866081	2.49E-08	3.39E-06
H2-T23	ENSMUSG00000067212.8	6.972569596	0.409722133	2.67E-08	3.59E-06
Oas1g	ENSMUSG00000066861.14	5.174121287	0.841759777	2.72E-08	3.62E-06
Ahnak	ENSMUSG00000069833.12	11.38379046	0.206470483	2.98E-08	3.93E-06
Gbp9	ENSMUSG00000029298.15	5.24083006	1.088114587	3.69E-08	4.82E-06
Rnf114	ENSMUSG00000006418.17	6.956325561	0.428380636	5.17E-08	6.69E-06
Ifi209	ENSMUSG00000043263.13	5.845208868	1.672433039	5.34E-08	6.84E-06
Itgb7	ENSMUSG00000001281.9	7.856607801	0.270076254	5.75E-08	7.29E-06
Phf11b	ENSMUSG00000091649.3	4.998290448	1.293530181	5.92E-08	7.43E-06
Gm42418	ENSMUSG00000098178.1	13.08503421	0.579995902	7.44E-08	9.25E-06
Shisa5	ENSMUSG00000025647.16	8.426622747	0.213260279	8.38E-08	1.03E-05
Axl	ENSMUSG00000002602.16	8.43535238	0.266456007	1.07E-07	1.31E-05
Slc11a1	ENSMUSG00000026177.11	7.639017386	-0.281974396	1.15E-07	1.38E-05
Gm23935	ENSMUSG00000076258.1	10.41301997	0.627685214	1.20E-07	1.43E-05
Actb	ENSMUSG00000029580.14	12.53627867	0.068950083	1.21E-07	1.44E-05
Gm8995	ENSMUSG00000063286.7	8.941469781	0.662849967	1.30E-07	1.53E-05
Man2b1	ENSMUSG00000005142.10	8.933930542	-0.201310803	1.36E-07	1.58E-05
Atp1a3	ENSMUSG00000040907.15	7.830610047	-0.259626623	1.39E-07	1.60E-05
Lpl	ENSMUSG00000015568.15	10.52425313	-0.111881383	1.42E-07	1.63E-05
Tapbp	ENSMUSG00000024308.14	8.712516408	0.308194095	1.48E-07	1.67E-05
Ascc3	ENSMUSG00000038774.8	6.817518834	0.390138648	1.64E-07	1.85E-05
Klhl24	ENSMUSG00000062901.2	5.38775785	-0.501551113	1.67E-07	1.86E-05
Nes	ENSMUSG00000004891.16	6.328747756	0.413776323	1.71E-07	1.89E-05
Oasl1	ENSMUSG00000041827.15	5.373382086	1.574068772	2.38E-07	2.61E-05
Cd47	ENSMUSG00000055447.18	8.366647896	0.247586783	2.41E-07	2.62E-05
Ccr5	ENSMUSG00000079227.9	5.995757282	0.576896024	2.44E-07	2.62E-05
Parp10	ENSMUSG00000063268.12	6.146948172	0.574236973	2.72E-07	2.90E-05
Tor1aip2	ENSMUSG00000050565.16	8.594931313	0.265634792	2.74E-07	2.91E-05
Gbp7	ENSMUSG00000040253.15	5.818150917	1.216359651	2.81E-07	2.95E-05
Tgtp2	ENSMUSG00000078921.3	4.793769711	1.727108983	2.89E-07	3.02E-05
H3f3b	ENSMUSG00000016559.14	9.568928743	0.177064117	3.12E-07	3.22E-05
Lars2	ENSMUSG00000035202.7	9.890138495	0.644412816	3.41E-07	3.50E-05
Mir6236	ENSMUSG00000098973.1	7.066669804	0.73781009	3.91E-07	3.95E-05
Sort1	ENSMUSG00000068747.14	6.712652208	-0.350462558	3.91E-07	3.95E-05

Dck	ENSMUSG00000029366.10	7.63754678	0.375991201	4.46E-07	4.47E-05
Irgm2	ENSMUSG00000069874.7	7.072108961	0.883950951	4.76E-07	4.73E-05
Cd34	ENSMUSG00000016494.9	7.256346547	0.291183672	5.51E-07	5.44E-05
Ifih1	ENSMUSG00000026896.14	7.145366879	0.787446967	6.36E-07	6.23E-05
Tor1aip1	ENSMUSG00000026466.16	8.153180672	0.314419496	6.54E-07	6.35E-05
Gclm	ENSMUSG00000028124.15	8.67300634	-0.185192957	6.64E-07	6.40E-05
Soat1	ENSMUSG00000026600.12	8.34698391	-0.208562808	6.75E-07	6.46E-05
Apobec3	ENSMUSG00000009585.17	6.643970354	0.454408417	7.94E-07	7.54E-05
Gm20559	ENSMUSG00000106734.3	6.65091584	0.505599741	8.89E-07	8.39E-05
Slc40a1	ENSMUSG00000025993.10	6.297251619	-0.393720796	9.54E-07	8.93E-05
Flrt2	ENSMUSG00000047414.6	6.188384413	0.476137838	1.00E-06	9.26E-05
Pnp	ENSMUSG00000021871.17	8.082629198	0.382517096	9.97E-07	9.26E-05
Vat1	ENSMUSG00000034993.7	9.408633229	-0.149053782	1.01E-06	9.26E-05
Igtp	ENSMUSG00000078853.8	6.135515	0.896262929	1.07E-06	9.72E-05
Tmsb10	ENSMUSG00000079523.8	8.178961916	0.214079338	1.18E-06	0.000106222
Dab2	ENSMUSG00000022150.16	9.600551159	0.115144422	1.32E-06	0.000118303
Ttyh2	ENSMUSG00000034714.9	6.386064898	-0.331253854	1.44E-06	0.000128162
Mov10	ENSMUSG00000002227.15	5.861067893	0.670577859	1.48E-06	0.000130604
Rxra	ENSMUSG00000015846.14	5.342063056	-0.392765125	1.61E-06	0.000141612
Rnf34	ENSMUSG00000029474.7	6.009923677	0.472036063	1.79E-06	0.000156003
H2-T22	ENSMUSG00000056116.18	6.614443515	0.556945575	1.97E-06	0.000170088
Ass1	ENSMUSG00000076441.9	6.002635459	0.442746033	1.96E-06	0.000170088
Mitd1	ENSMUSG00000026088.15	4.9520863	0.735165997	2.00E-06	0.000171443
Ccnd2	ENSMUSG00000000184.12	7.34069694	0.353394665	2.63E-06	0.000223874
Psme1	ENSMUSG00000022216.16	6.971824919	0.324427133	2.83E-06	0.000238809
Gas6	ENSMUSG00000031451.6	8.628836782	-0.172138618	3.06E-06	0.000256551
Plec	ENSMUSG00000022565.15	10.44195699	0.16089927	3.36E-06	0.000280085
Atp13a3	ENSMUSG00000022533.13	7.981018663	-0.214835173	3.53E-06	0.00029265
Phf11d	ENSMUSG00000068245.14	4.679659567	0.733243656	3.74E-06	0.000305853
Ms4a6c	ENSMUSG00000079419.4	7.310095946	0.342893931	3.72E-06	0.000305853
Mx1	ENSMUSG00000000386.14	6.586672035	2.030930638	4.04E-06	0.000326731
Herc6	ENSMUSG00000029798.11	5.489313997	0.771185187	4.05E-06	0.000326731
Lamp1	ENSMUSG00000031447.7	10.84182726	-0.065874828	4.14E-06	0.00033245
Ifi206	ENSMUSG00000037849.7	3.866211909	1.88268846	4.42E-06	0.00035287
Gm12250	ENSMUSG00000082292.3	5.434838562	1.217368859	4.51E-06	0.000357795
Aplp2	ENSMUSG00000031996.16	8.581317871	-0.174456818	5.13E-06	0.000404043
Smpdl3a	ENSMUSG00000019872.13	8.176942787	-0.170702581	5.60E-06	0.000438494
Trim14	ENSMUSG00000039853.18	5.343926752	0.573760985	6.07E-06	0.000472368
Aldh2	ENSMUSG00000029455.14	9.11369646	-0.140833423	6.14E-06	0.000474823
Gm5431	ENSMUSG00000058163.13	5.582780563	0.967672313	6.31E-06	0.000485616
Ccr1	ENSMUSG00000025804.4	4.454349722	0.567031785	6.81E-06	0.000517652
Man1c1	ENSMUSG00000037306.13	7.251245294	-0.232339735	6.81E-06	0.000517652
Gnas	ENSMUSG00000027523.19	9.579411952	-0.115630655	6.95E-06	0.000525045
Atp6v1a	ENSMUSG00000052459.13	9.60564893	-0.11527043	7.04E-06	0.000529027
Pik3ap1	ENSMUSG00000025017.9	8.709219657	0.170276095	7.11E-06	0.000530824

Chmp4b	ENSMUSG00000038467.15	7.961209469	0.203960077	7.42E-06	0.000550622
Abcd2	ENSMUSG00000055782.8	3.004724609	-0.955587175	8.03E-06	0.000592546
Gm4070	ENSMUSG00000078606.8	4.26808386	1.120729383	9.52E-06	0.00069894
Gbp3	ENSMUSG00000028268.14	5.831271482	1.188753262	1.01E-05	0.000733641
Cd300lf	ENSMUSG00000047798.15	6.448209986	0.311259307	1.01E-05	0.000733641
Ifit1	ENSMUSG00000034459.8	7.871835625	1.463500997	1.15E-05	0.000831026
Man2a2	ENSMUSG00000038886.10	5.89285581	-0.348905533	1.28E-05	0.000921206
Tnfrsf21	ENSMUSG00000023915.4	5.518444637	-0.369315184	1.35E-05	0.000962987
Cfp	ENSMUSG00000001128.7	8.022278279	0.183089601	1.40E-05	0.000996157
Aim1	ENSMUSG00000019866.13	7.197652088	0.310003729	1.43E-05	0.001011401
Pls3	ENSMUSG00000016382.15	7.376615979	0.232519836	1.47E-05	0.001033047
Malat1	ENSMUSG00000092341.2	8.906548362	0.222403646	1.53E-05	0.001069949
Hmox1	ENSMUSG00000005413.7	9.665426472	-0.134679272	1.54E-05	0.001069949
Usp25	ENSMUSG00000022867.9	8.30776116	0.215171355	1.57E-05	0.0010868
Flna	ENSMUSG00000031328.15	11.46756123	0.073976498	1.60E-05	0.00109874
Ctsa	ENSMUSG00000017760.15	9.688463517	-0.096513478	1.62E-05	0.00109874
Galc	ENSMUSG00000021003.9	6.550808657	-0.306459224	1.62E-05	0.00109874
Itpr1	ENSMUSG00000030102.10	5.532356697	0.385480407	1.64E-05	0.001109703
Parp11	ENSMUSG00000037997.12	5.388831206	0.65804449	1.67E-05	0.001125227
Hpse	ENSMUSG00000035273.14	6.545556642	-0.311890116	1.80E-05	0.001204915
Ifit3b	ENSMUSG00000062488.9	5.286860423	1.731131055	1.85E-05	0.001228989
Tpst1	ENSMUSG00000034118.15	5.196265944	0.587444553	2.00E-05	0.001326041
Lgals8	ENSMUSG00000057554.13	7.226568699	0.249043789	2.02E-05	0.001328318
Actg1	ENSMUSG00000062825.15	9.746071696	0.103117904	2.19E-05	0.001438072
Azi2	ENSMUSG00000039285.12	6.564444241	0.322988136	2.28E-05	0.001485778
Agrn	ENSMUSG00000041936.18	4.970070358	0.590771965	2.38E-05	0.001546368
Pgap1	ENSMUSG00000073678.4	5.592158556	-0.397683809	2.46E-05	0.001591167
Ifit2	ENSMUSG00000045932.12	8.619484265	1.419376549	2.54E-05	0.00163445
Tbc1d16	ENSMUSG00000039976.4	4.720325617	-0.4422659	2.62E-05	0.001675165
Hexa	ENSMUSG00000025232.7	9.4573004	-0.106518808	2.73E-05	0.001736067
Uba7	ENSMUSG00000032596.14	5.985515715	0.445422308	3.06E-05	0.001937255
H2-Q6	ENSMUSG00000073409.12	2.372116923	1.779632796	3.15E-05	0.001985318
Tcof1	ENSMUSG00000024613.16	7.136090355	0.254984875	3.90E-05	0.002448066
Ddx24	ENSMUSG00000041645.13	7.19371927	0.249138721	4.30E-05	0.0026822
Cspg4	ENSMUSG00000032911.5	7.368719112	0.204289895	4.36E-05	0.002710304
Zmat3	ENSMUSG00000027663.12	5.898007922	-0.371668433	4.40E-05	0.002719556
Ms4a4c	ENSMUSG00000024675.19	3.679752586	1.519679652	4.94E-05	0.003022038
C5ar1	ENSMUSG00000049130.6	8.074544047	0.151610567	4.93E-05	0.003022038
Abi2	ENSMUSG00000026782.15	5.226306571	-0.426062498	4.96E-05	0.003023474
Trim34a	ENSMUSG00000056144.14	5.295050457	0.583544133	5.09E-05	0.003073601
Cd28	ENSMUSG00000026012.2	6.81555093	-0.2445353	5.08E-05	0.003073601
Anxa1	ENSMUSG00000024659.14	9.542263534	0.106321397	5.46E-05	0.00328383
Cox15	ENSMUSG00000040018.9	5.761135557	0.351467397	5.52E-05	0.003298811
Trim12c	ENSMUSG00000057143.15	5.636958295	0.44151261	5.68E-05	0.003380755
Endod1	ENSMUSG00000037419.8	7.259040936	0.242852464	5.89E-05	0.003473101

Actr3	ENSMUSG00000026341.16	9.666839507	0.10483042	5.87E-05	0.003473101
Mthfr	ENSMUSG00000029009.17	6.729846393	0.340628965	6.02E-05	0.003537526
Atp6ap1	ENSMUSG00000019087.13	8.710732277	-0.130293173	6.07E-05	0.003547799
Ccr2	ENSMUSG00000049103.13	5.060468439	0.409224905	6.58E-05	0.003828218
Ehd1	ENSMUSG00000024772.9	8.664402339	0.181995771	6.79E-05	0.003932706
Myh9	ENSMUSG00000022443.16	10.70851511	0.078555633	6.90E-05	0.003982229
Gm15564	ENSMUSG00000086324.8	4.52255344	0.792670543	7.03E-05	0.004019473
Lrrk2	ENSMUSG00000036273.15	3.031665985	-0.891070094	7.03E-05	0.004019473
Gm24270	ENSMUSG00000076281.1	5.751627235	0.621161962	7.25E-05	0.004126754
Psme2	ENSMUSG00000079197.10	7.173102155	0.267449499	7.34E-05	0.004139286
Cd68	ENSMUSG00000018774.13	10.33303479	-0.081510313	7.33E-05	0.004139286
Fus	ENSMUSG00000030795.18	8.452681366	0.145846739	7.44E-05	0.004180114
Gstm1	ENSMUSG00000058135.12	6.697788264	-0.262379316	7.47E-05	0.004180114
Gusb	ENSMUSG00000025534.17	9.699470422	-0.092590018	7.62E-05	0.00422721
Creg1	ENSMUSG00000040713.12	9.287595953	-0.111597842	7.59E-05	0.00422721
Sirpa	ENSMUSG00000037902.18	10.48536969	-0.075455819	7.99E-05	0.004410988
Psmb10	ENSMUSG00000031897.8	6.267466534	0.399684647	8.67E-05	0.004767729
Mdh1	ENSMUSG00000020321.15	7.410318475	-0.178835527	8.96E-05	0.004907813
Msh2	ENSMUSG00000024151.13	4.35588844	-0.48646541	9.57E-05	0.00521905
Nfkbiz	ENSMUSG00000035356.16	5.594044002	0.715925024	9.67E-05	0.005248609
Ucp2	ENSMUSG00000033685.13	9.04500205	-0.111059125	0.000100562	0.005436265
Pml	ENSMUSG00000036986.16	6.580216166	0.437602421	0.000103588	0.005576498
Vsir	ENSMUSG00000020101.14	7.088051305	-0.186398018	0.000114225	0.006123578
Mx2	ENSMUSG00000023341.14	4.917953147	1.451196149	0.000115324	0.006156982
Clec4b1	ENSMUSG00000030147.12	-0.415701325	2.706488357	0.000116934	0.006217233
Lmna	ENSMUSG00000028063.15	8.986354966	0.113013293	0.000118373	0.006267964
Tmem229b	ENSMUSG00000046157.13	6.60149323	0.372658195	0.000123908	0.006534233
Hspa5	ENSMUSG00000026864.13	9.982381404	0.092810646	0.000148499	0.00779922
Tcn2	ENSMUSG00000020432.12	6.510712663	-0.267635669	0.000153434	0.008025802
Ifi44	ENSMUSG00000028037.13	3.272911906	1.485210637	0.00015954	0.008311532
Ccl7	ENSMUSG00000035373.2	7.252911127	0.440369118	0.000165724	0.008599011
Gm6548	ENSMUSG00000091549.1	5.214359085	0.463745319	0.000166469	0.008603112
Ephx1	ENSMUSG00000038776.13	3.721064373	-0.583404955	0.000173323	0.008921624
Snx2	ENSMUSG00000034484.7	8.31431426	0.150751209	0.000176605	0.009054516
H2-Aa	ENSMUSG00000036594.14	5.002334276	0.442817675	0.000185066	0.009450813
Pgm2l1	ENSMUSG00000030729.17	6.388239702	-0.239739248	0.000189575	0.009642972
Fbxw17	ENSMUSG00000037816.10	4.780004708	0.534197083	0.00019154	0.009704679
Ubr4	ENSMUSG00000066036.14	8.903915301	0.171035448	0.000195306	0.009833584
Tagln2	ENSMUSG00000026547.15	9.354831629	0.097447722	0.000195606	0.009833584
Mef2a	ENSMUSG00000030557.17	8.769870803	0.098686681	0.000203552	0.010193389
Lin7c	ENSMUSG00000027162.7	7.02577811	-0.219396043	0.000213547	0.010652621
Cd72	ENSMUSG00000028459.11	7.309072661	0.1948215	0.000216834	0.010774977
Pam	ENSMUSG00000026335.16	6.553713756	-0.238447071	0.000218324	0.010807444
Capza2	ENSMUSG00000015733.13	8.971347719	0.118118145	0.000220948	0.010895622

Gm24187	ENSMUSG00000088609.1	4.814786195	0.603072261	0.000222666	0.010922971
Fam46a	ENSMUSG00000032265.14	6.476493221	0.443625813	0.000223194	0.010922971
Mospd2	ENSMUSG00000061778.10	5.963937122	-0.288860164	0.000224279	0.010934659
Ptgfrn	ENSMUSG00000027864.9	3.330474778	-0.627491943	0.000226222	0.010987918
Daxx	ENSMUSG00000002307.15	7.068496525	0.469871957	0.000227532	0.01101018
D17Wsu92e	ENSMUSG00000056692.12	7.605248549	0.178288883	0.000232709	0.011218662
Kcnj2	ENSMUSG00000041695.2	4.787619288	-0.449024113	0.000233815	0.011230091
Plbd1	ENSMUSG00000030214.6	0.059934081	1.860663173	0.000247812	0.011858256
Pmp22	ENSMUSG00000018217.12	8.735812054	0.121869671	0.000263732	0.01257349
Cd63	ENSMUSG00000025351.13	8.819503259	-0.115205765	0.000273953	0.0129651
Gaa	ENSMUSG00000025579.14	6.742421014	-0.219902196	0.000273372	0.0129651
Neat1	ENSMUSG00000092274.2	6.422114813	0.234926169	0.000285262	0.013451036
Olfr56	ENSMUSG00000040328.14	3.382324634	2.010527026	0.000289353	0.013594326
Gbp2	ENSMUSG00000028270.12	6.549842446	1.146748526	0.000298158	0.013789746
H2-Eb1	ENSMUSG00000060586.10	4.130272436	0.643793726	0.000297139	0.013789746
Tap2	ENSMUSG00000024339.12	6.56045967	0.329188623	0.000295246	0.013789746
Tdrd7	ENSMUSG00000035517.17	6.660773007	0.26298296	0.000296405	0.013789746
S100a10	ENSMUSG00000041959.14	7.726323853	0.185315778	0.000298849	0.013789746
Ugp2	ENSMUSG00000001891.16	7.296368117	-0.18988942	0.000301932	0.013833212
Clstn1	ENSMUSG00000039953.13	3.352338829	-0.707411962	0.000301655	0.013833212
Bhlhe41	ENSMUSG00000030256.11	6.388972775	-0.227370165	0.00030556	0.013949926
Gvin1	ENSMUSG00000045868.12	4.14403902	1.079164896	0.000309131	0.014063266
St3gal2	ENSMUSG00000031749.12	5.325923305	-0.311600668	0.000311323	0.014113326
Gas7	ENSMUSG00000033066.15	7.348269087	0.219976362	0.000320936	0.014397536
Dtx4	ENSMUSG00000039982.7	7.160062895	-0.177790888	0.000320501	0.014397536
Ldhb	ENSMUSG00000030246.11	1.604332372	-1.377430425	0.000320221	0.014397536
Gch1	ENSMUSG00000037580.9	5.825996047	0.405969659	0.000324572	0.014510271
Dapk1	ENSMUSG00000021559.13	7.205381922	-0.211964796	0.000338221	0.015068327
Dync1h1	ENSMUSG00000018707.13	9.955740576	0.122752047	0.000341678	0.015170013
Cadm1	ENSMUSG00000032076.18	8.872840605	0.125287962	0.000349129	0.015447777
Tnfrsf11a	ENSMUSG00000026321.7	7.13573829	0.179501067	0.000364549	0.016074998
Ulk1	ENSMUSG00000029512.11	5.100322694	-0.360664095	0.000366241	0.016094679
Skil	ENSMUSG00000027660.16	8.697422933	0.129878507	0.000374672	0.016409346
Sh3pxd2a	ENSMUSG00000053617.11	5.951943933	-0.308580554	0.000381709	0.016661095
Hexb	ENSMUSG00000021665.7	8.680552816	-0.116480009	0.000392302	0.016909869
Plxnd1	ENSMUSG00000030123.15	8.593755949	-0.139221854	0.000392644	0.016909869
Fabp4	ENSMUSG00000062515.3	3.781140353	-0.669775243	0.000389353	0.016909869
Rtn4r	ENSMUSG00000043811.5	-0.889083342	-2.85770211	0.000391865	0.016909869
Gdf15	ENSMUSG00000038508.7	5.298198962	-0.450634626	0.000398482	0.017104289
Txnrd1	ENSMUSG00000020250.9	9.760545052	-0.095398138	0.000406856	0.017405876
Gpx3	ENSMUSG00000018339.11	4.746030693	-0.378894749	0.000408456	0.017416668
Rmnd5a	ENSMUSG00000002222.14	6.366290655	-0.250931013	0.000411518	0.017489502
Mrpl1	ENSMUSG00000029486.13	4.643434684	0.403797834	0.000423158	0.017925247
Psmb9	ENSMUSG00000096727.2	5.428088172	0.423868119	0.000429471	0.018133231

Ftl1	ENSMUSG00000050708.16	12.08182854	-0.056821995	0.000430985	0.018137876
Alcam	ENSMUSG00000022636.13	9.282409416	-0.100229961	0.00044173	0.018529716
Mpc1	ENSMUSG00000023861.17	5.095516869	-0.384299648	0.000452667	0.018927049
Anpep	ENSMUSG00000039062.15	10.09729652	-0.072257213	0.000456062	0.019007472
Xlr	ENSMUSG00000054626.11	0.443153496	-1.779512383	0.000464332	0.019289954
Irf5	ENSMUSG00000029771.12	8.11149574	0.151067666	0.000470639	0.019489285
Emilin2	ENSMUSG00000024053.10	9.514764419	0.089387325	0.000487549	0.020125019
Gm21781	ENSMUSG00000095123.1	1.572675662	1.087364445	0.000491792	0.020171263
Clic4	ENSMUSG00000037242.8	9.877689435	0.121327992	0.000490398	0.020171263
Sgpl1	ENSMUSG00000020097.14	9.197811232	-0.094833278	0.000495535	0.020260497
Cd9	ENSMUSG00000030342.8	8.881603307	-0.106052029	0.000508914	0.020696117
Gna12	ENSMUSG00000000149.10	7.504430378	-0.164315726	0.000509394	0.020696117
Phf11a	ENSMUSG00000044703.5	2.042851144	1.730478642	0.000512092	0.020740547
Rasa4	ENSMUSG00000004952.13	7.115252245	0.220496516	0.000516376	0.020848682
Cmpk2	ENSMUSG00000020638.7	7.913946341	1.383980312	0.000527287	0.021222908
Adipor1	ENSMUSG00000026457.14	7.421787999	-0.169500363	0.000531825	0.021339077
Pou3f1	ENSMUSG00000090125.3	1.672549442	2.343827663	0.000541369	0.021654768
Nr2f6	ENSMUSG00000002393.14	3.516098986	-0.555939543	0.000544174	0.021699762
Rap1gap	ENSMUSG00000041351.16	-0.667347062	-2.5160717	0.000549325	0.021837792
Nat8l	ENSMUSG00000048142.9	0.597314897	-1.586712392	0.000565453	0.022409978
Eps8	ENSMUSG00000015766.14	4.850992363	0.423333171	0.000575289	0.022730054
Large1	ENSMUSG00000004383.17	5.238018145	-0.359590693	0.000588153	0.023167493
Tiam1	ENSMUSG00000002489.15	5.968170758	0.280458685	0.000592694	0.023275382
Btg1	ENSMUSG00000036478.7	6.709546051	0.229639783	0.000596374	0.023348931
Arl8b	ENSMUSG00000030105.8	8.262586581	-0.120565899	0.000608974	0.023698641
Pank3	ENSMUSG00000018846.8	7.369094458	-0.179909532	0.000608873	0.023698641
Rap1gds1	ENSMUSG00000028149.12	7.413105553	-0.148443946	0.000624783	0.024240844
Arhgap30	ENSMUSG00000048865.16	8.194344677	0.13367178	0.00063278	0.024331884
Pgk1	ENSMUSG00000062070.12	8.882278941	-0.102580091	0.000632291	0.024331884
Pea15a	ENSMUSG00000013698.12	7.955013114	-0.149668431	0.000630208	0.024331884
Edem1	ENSMUSG00000030104.9	8.823155416	-0.115964013	0.000645161	0.024734359
Sidt2	ENSMUSG00000034908.15	6.278046306	-0.240623721	0.000648814	0.024800818
Ankrd11	ENSMUSG00000035569.16	7.789820047	0.143827294	0.000655858	0.02499613
Abcb1b	ENSMUSG00000028970.9	6.83360199	-0.198880133	0.00065832	0.025016165
Gla	ENSMUSG00000031266.6	7.678625275	-0.143288579	0.000668136	0.025314726
BC005537	ENSMUSG00000019132.10	10.3730313	0.065070295	0.00067457	0.02548376
Pms1	ENSMUSG00000026098.13	2.776919165	0.775359062	0.000684796	0.025794656
Slc7a11	ENSMUSG00000027737.10	7.289424866	-0.289701674	0.00070115	0.026333897
Sdc4	ENSMUSG00000017009.3	8.086577771	0.176916984	0.000713619	0.026703865
Plxnc1	ENSMUSG00000074785.4	7.906572608	-0.135261987	0.000715134	0.026703865
Trim25	ENSMUSG00000000275.16	8.345331007	0.176013359	0.000722662	0.02690719
Htr2b	ENSMUSG00000026228.6	4.422473282	-0.376099646	0.000732966	0.027212431
Pla2g7	ENSMUSG00000023913.17	3.933082478	-0.548160829	0.000735352	0.027222789
Ubald1	ENSMUSG00000039568.6	4.734431405	-0.339887445	0.000752933	0.027793993

Csf2ra	ENSMUSG00000059326.6	6.772175662	-0.201380178	0.000760792	0.028004071
Tpp1	ENSMUSG00000030894.5	8.50815567	-0.121967265	0.000763214	0.028013434
Anxa3	ENSMUSG00000029484.12	7.737810293	-0.154520876	0.000766092	0.028039418
Nmi	ENSMUSG00000026946.9	4.935631986	0.488880018	0.000769749	0.028093676
Ifi213	ENSMUSG00000073491.10	2.584437787	1.74931211	0.000775276	0.028215688
Fxyd5	ENSMUSG00000009687.14	9.159678716	0.097338193	0.000787453	0.028504179
Tecpr1	ENSMUSG00000066621.12	7.455693569	-0.148849384	0.000787615	0.028504179
Nectin4	ENSMUSG00000006411.12	4.248372268	0.525432867	0.00079133	0.028558619
Tlr3	ENSMUSG00000031639.12	5.915808892	0.476508403	0.000812201	0.029230176
Olfm1	ENSMUSG00000026833.18	5.677163095	0.256674367	0.000827391	0.02969414
S100a11	ENSMUSG00000027907.4	6.196954065	0.289350182	0.000833529	0.029831551
Trp53inp2	ENSMUSG00000038375.15	6.484161169	-0.203284064	0.000858329	0.030634278
Ypel3	ENSMUSG00000042675.15	3.814228406	-0.438175717	0.000868772	0.030921596
Casp1	ENSMUSG00000025888.5	6.839552354	0.25418524	0.000878619	0.030931207
Dhrs9	ENSMUSG00000027068.6	6.113557385	0.252006542	0.000872049	0.030931207
Ccl9	ENSMUSG00000019122.8	9.765103921	0.123474321	0.000877755	0.030931207
Tgfb1	ENSMUSG00000002603.15	8.460681485	-0.115675778	0.000876242	0.030931207
Cdc42ep3	ENSMUSG00000036533.8	4.833544103	-0.344631178	0.000896442	0.031387614
Lyz1	ENSMUSG00000069515.5	0.874625307	-1.476494222	0.000894618	0.031387614
H1f0	ENSMUSG00000096210.1	6.345744749	0.200349213	0.000919069	0.032092896
Card11	ENSMUSG00000036526.8	5.409307545	0.305130465	0.000931248	0.03243053
Dmxl2	ENSMUSG00000041268.16	5.245370572	-0.318792847	0.00097127	0.033733365
Emc6	ENSMUSG00000047260.4	4.995296923	-0.350966523	0.000995938	0.03449738
Ctsl	ENSMUSG00000021477.8	9.506588398	0.078445306	0.001005225	0.034725943
Ubxn2a	ENSMUSG00000020634.12	4.916073081	-0.348754656	0.001020786	0.035169471
Hnrnpdl	ENSMUSG00000029328.15	7.563109439	0.166262737	0.00103679	0.035625878
Anxa5	ENSMUSG00000027712.13	9.321760786	-0.084551787	0.001057081	0.036226754
Lrp6	ENSMUSG00000030201.15	6.388507566	-0.191437381	0.001062062	0.03630115
Gpr132	ENSMUSG00000021298.7	4.22257557	0.593286061	0.001085629	0.03700879
Prdx1	ENSMUSG00000028691.12	10.64012634	-0.085223427	0.001107451	0.037653321
Nampt	ENSMUSG00000020572.7	7.995754488	0.22765446	0.001130848	0.038347914
Mgst1	ENSMUSG00000008540.11	4.071571397	-0.515451714	0.001138127	0.038493705
Trim5	ENSMUSG00000060441.15	3.086650707	0.731633149	0.001164912	0.039296761
Isg20	ENSMUSG00000039236.18	3.18996815	1.307800955	0.001173229	0.039474281
Gpr137b	ENSMUSG00000021306.10	8.167378954	-0.137668992	0.001187391	0.039846985
Tsku	ENSMUSG00000049580.12	2.240871047	-0.94397223	0.001209603	0.040382614
3830403N18Rik	ENSMUSG00000031125.2	0.363555276	-2.001336758	0.001208838	0.040382614
Tgfbr1	ENSMUSG00000007613.15	8.536445582	-0.106799587	0.001223792	0.040751022
Rtn4	ENSMUSG00000020458.16	9.904922545	-0.072029247	0.001255304	0.04169286
Mertk	ENSMUSG00000014361.5	4.581501973	-0.314930372	0.001269898	0.042069431
Atg5	ENSMUSG00000038160.6	5.396938441	-0.289093996	0.001278927	0.042260199
Cd109	ENSMUSG00000046186.8	6.787624016	0.206373073	0.001284462	0.042334831
Myo5a	ENSMUSG00000034593.16	9.070475228	-0.101493241	0.001309924	0.043064173
Mlkl	ENSMUSG00000012519.14	5.807586901	0.330834945	0.001321365	0.043330044
Max	ENSMUSG00000059436.12	6.663783154	0.206252255	0.001390639	0.045486209

Syne3	ENSMUSG00000054150.11	6.518781749	0.192699689	0.001399018	0.045644728
Cyfip2	ENSMUSG00000020340.16	7.974446945	-0.125861413	0.001435312	0.046710906
Ipo13	ENSMUSG00000033365.14	6.10597547	-0.225945188	0.001445915	0.046937756
Lipa	ENSMUSG00000024781.15	10.72175353	-0.06096717	0.001464654	0.047426901
Pa2g4	ENSMUSG00000025364.12	7.370198152	0.157371916	0.001490067	0.047889709
Laptm5	ENSMUSG00000028581.17	10.51331308	-0.049321754	0.001488315	0.047889709
Tsen34	ENSMUSG00000035585.16	4.748535491	-0.326014998	0.001487566	0.047889709
BC022960	ENSMUSG00000081137.3	-0.268073371	1.805322054	0.001506036	0.048282839
9130208D14Rik	ENSMUSG00000086513.3	-0.132787134	1.888593018	0.001524272	0.048626148
Dynll2	ENSMUSG00000020483.14	6.846019927	-0.17508606	0.001522874	0.048626148
Blvrb	ENSMUSG00000040466.16	6.741293178	-0.186390136	0.001541835	0.049065296
Plek	ENSMUSG00000020120.15	9.890259281	-0.111998424	0.001550989	0.049112521
Slc38a7	ENSMUSG00000036534.5	6.126814397	-0.244800822	0.001554246	0.049112521
Tmem115	ENSMUSG00000010045.2	5.237195689	-0.300142695	0.001554723	0.049112521
Adss	ENSMUSG00000015961.8	7.379138278	0.156517401	0.001559145	0.049132076

*Major significantly upregulated or downregulated genes in *Lrrk2* KO macrophages compared to *Lrrk2* HET controls. Significant genes are based on adjusted p value. Table is arranged by adjusted p value low>high values.
